# Recommendations for implementing patient blood management—an updated modified Delphi consensus from a multidisciplinary expert panel in Hong Kong

**DOI:** 10.1186/s44158-026-00417-4

**Published:** 2026-05-26

**Authors:** Hung-Kai Cheng, Kin-Chung Tse, Luke Ka-Lok Chan, Martin Tsang-Tung Chan, Benny Chun-Pong Cheng, Carmen Ka-Man Choi, Yu-Fat Chow, Cheuk-Kwong Lee, Wing-Cheong Leung, Tsin-Wah Leung, Simmy Ming-Wai Sin, Rita Ching-Yee So, Steven Ho-Shan Wong

**Affiliations:** 1 Department of Anaesthesia and Operating Theatre Services, Tseung Kwan O Hospital, Tseung Kwan O, Hong Kong; 2 Division of Haematology and Haematological Oncology, Department of Medicine and Geriatrics, Princess Margaret Hospital, Kwai Chung, Hong Kong; 3 Department of Orthopaedics and Traumatology, Tseung Kwan O Hospital, Tseung Kwan O, Hong Kong; 4 Department of Anaesthesia and Operating Theatre Services, Tuen Mun Hospital; Pok Oi Hospital; and Tin Shui Wai Hospital, New Territories West, Hong Kong; 5 Department of Obstetrics and Gynaecology, Tseung Kwan O Hospital, Tseung Kwan O, Hong Kong; 6 Department of Anaesthesiology, Hong Kong Sanatorium & Hospital, Happy Valley, Hong Kong; 7 Hong Kong Red Cross Blood Transfusion Service, Yau Tsim Mong, Hong Kong; 8 Department of Obstetrics and Gynaecology, Kwong Wah Hospital, Yau Ma Tei, Hong Kong; 9 Department of Anaesthesia, Princess Margaret Hospital; North Lantau Hospital; and Yan Chai Hospital, Kowloon West, Hong Kong; 10 Department of Anaesthesiology, Perioperative and Pain Medicine, Queen Elizabeth Hospital, Kowloon, Hong Kong

**Keywords:** Anaemia, Haemostasis, Iron deficiency, Postpartum haemorrhage, Prothrombin complex concentrate, Thromboelastography

## Abstract

In 2020, the Hong Kong Society of Clinical Blood Management (HKSCBM) established a consensus statement on the implementation of patient blood management (PBM). Based on recent evidence on PBM initiatives and practices, the HKSCBM updated their consensus statements. HKSCBM convened a multidisciplinary panel of 13 experts, who formulated discussion questions across three PBM pillars: (1) optimising patients’ red blood cell mass and improving anaemia management; (2) minimising perioperative blood loss; and (3) rationalising the use of blood and blood components. Using the modified Delphi method, the panel addressed the discussion questions by reviewing relevant literature and clinical experiences in multiple meetings. Consensus statements derived from meeting proceedings were voted anonymously by the panel. A consensus statement was accepted only if ≥ 80% of the panel chose ‘accept completely’ or ‘accept with some reservation’, based on a 5-point Likert scale. The panel accepted 117 consensus statements (including sub-statements). The updated version includes new additions to the following areas: identification of groups at high risk for iron deficiency anaemia; preoperative screening and management of iron deficiency and/or anaemia; recognition of bleeding-prone surgeries and conditions; recent measures to minimise intraoperative blood loss, including point-of-care coagulation testing, tranexamic acid, fibrinogen concentrate, reversal agents for anticoagulation, advanced haemodynamic monitoring, and uterotonics; suggested haemoglobin thresholds for transfusions; and implementation of transfusion protocols. These consensus statements are intended to serve as a practical framework for medical and surgical colleagues in Hong Kong and the Asia–Pacific region.

**Supplementary Information:**

The online version contains supplementary material available at 10.1186/s44158-026-00417-4.

## Introduction

Patient blood management (PBM) represents a comprehensive approach for optimising patient outcomes and safety through a range of screening, medical, and surgical measures to manage iron deficiency with or without anaemia, coagulopathy, and at the same time preserve the usage of blood components [1, 2]. Endorsed by the World Health Organisation (WHO), the notion of PBM has gained substantial traction globally through growing awareness of the risk of transfusion-related adverse effects, including infection, prolonged hospitalisation, and morbimortality, alongside considerable healthcare costs [3]. In 2020, the Hong Kong Society of Clinical Blood Management (HKSCBM) established a consensus statement to facilitate the implementation of PBM in the locality [4]. With the continuous development of PBM initiatives, the HKSCBM issued an updated set of consensus statements on the basis of recent evidence. Because the evidence base regarding PBM remains limited, heterogeneous, and in some areas conflicting, it was considered insufficient to support a formal systematic review or evidence-based guideline development process. Therefore, a consensus approach was deemed the most appropriate methodology to address the topic, integrating the available literature with expert insights, clinical experience, and practical considerations relevant to current practice. Although this consensus protocol was not prospectively registered, its reporting aligned with the ACCORD guidelines (Additional file 1) [5].

## Method

The president and vice-president of the HKSCBM served as co-chairs responsible for convening a consensus panel of 11 Society members who were invited on the basis of diverse special interests and extensive clinical experience (> 10 years) in medical or surgical aspects of PBM. These 13 panellists comprised six anaesthesiologists (including both co-chairs), three specialists in obstetrics and gynaecology, two specialists in internal medicine, haematology and haematological oncology, and transfusion medicine, one specialist in orthopaedics and traumatology, and one anaesthetic nurse, most of whom practised in the public healthcare sector. Compared with the previous consensus panel of 11 members predominantly from anaesthesiology, an expansion of the number and specialty of panellists was expected to address recent developments across diverse PBM practices. The panel’s composition reflected the practical application of PBM practices in Hong Kong’s clinical environment, where most patients receive care in public hospitals, with anaesthesiologists typically assuming a leadership role while other specialists participate to varying extents.

Prior to consensus meetings, the co-chairs formulated discussion questions based on three pillars of PBM: (1) optimising patients’ red blood cell (RBC) mass and improving anaemia management; (2) minimising perioperative blood loss; and (3) rationalising the use of blood and blood components.

With the aim of providing a relatively rapid and broad overview of the topic to contextualise panel discussions, the consensus panel commissioned a medical communications agency to assist with a narrative rather than systematic literature search. The PubMed database was searched for meta-analyses, randomised controlled trials, observational studies, reviews, and clinical guidelines that addressed PBM-related areas for the publication period between June 2020 and June 2025, using the following keywords: ‘anaemia’; ‘fibrinogen’; ‘haemodynamic monitoring’; ‘haemostatic agent’; ‘intravenous iron’; ‘iron deficiency’; ‘nurse-led clinic’; ‘oral iron’; ‘postpartum haemorrhage’; ‘restrictive transfusion’; ‘single-unit transfusion’; ‘thromboelastography’; ‘tranexamic acid’; ‘transfusion threshold’; and ‘transfusion trigger’. The titles and abstracts of the search results were screened to identify relevant papers for discussion.

Using the modified Delphi method [6], the panel formulated consensus statements through a series of discussions and votes. Based on personal interest and expertise, three subgroups were formed to address the discussion areas. In three online meetings held on 8th September, 22nd September, and 15th October 2025, the panellists took a turn to present the relevant literature and their clinical experiences regarding their designated discussion questions, followed by panel discussions and comments. These meetings familiarised all panellists with all discussion areas addressed by each of them and facilitated the subsequent statement drafting and voting processes. Draft consensus statements, prepared by the medical communications agency based on the meeting proceedings, were circulated among panellists via a messaging platform. The panel suggested adding, removing, merging, and revising statements where appropriate. All panellists participated in the review, revision, and voting processes for the final consensus statements.

At a physical meeting held on 21 st November 2025, an updated set of statements was discussed, revised, and finalised, followed by anonymous panel voting (including co-chairs) via an electronic system. A consensus statement was accepted only if ≥ 80% of the panel chose ‘accept completely’ or ‘accept with some reservation’, based on a 5-point Likert scale; the other options were ‘accept with major reservation’, ‘reject with reservation’, and ‘reject completely’.

On 9th December 2025, the panel reviewed the voting results in an online meeting. Among a total of 118 statements (including sub-statements), 114 and four were accepted and rejected, respectively. The wording of the accepted statements remained unchanged. For the remaining four, one was rejected because its suggestion (an optimal preoperative haemoglobin (Hb) level ≥ 13.0 g/dL in both males and females) was overridden by another (≥ 13.0 g/dL in males and ≥ 12.0 g/dL in females). The other three rejected statements were modified and eventually accepted through anonymous online voting. Additional file 2 reports the final voting records.

## Results

The panel accepted 117 consensus statements, of which 29 were chosen with a vote of ‘accept completely’ by < 80% of panellists (Table 1). The rationale for each statement is discussed below. The updated version includes additions to a number of important areas, as follows: identification of groups at high risk for iron deficiency anaemia (IDA); preoperative screening and management of iron deficiency and/or anaemia among general and special surgical populations; recognition of bleeding-prone surgeries and conditions; recent measures to minimise intraoperative blood loss, including point-of-care (POC) coagulation testing, tranexamic acid (TXA), fibrinogen concentrate, reversal agents for anticoagulation, advanced haemodynamic monitoring, and uterotonics for postpartum haemorrhage (PPH); suggested Hb thresholds for transfusions among different surgical populations; and implementation of transfusion protocols. Although the literature search was cut off in June 2025, the panel noted that, to the best of their knowledge, no studies published between July 2025 and April 2026 substantially impacted the established statements.

**Table 1:** All accepted consensus statements

		% of panellists (*N* = 13)	
#	**Accepted statements**	**Accept completely**	**Accept with some reservation**
**Part 1. Optimising patients’ red blood cell mass and improving anaemia management**			
1.1. Groups at high risk for iron deficiency and anaemia			
1	Groups at high risk for iron deficiency and anaemia include (Table 2):		
1a	Women at reproductive age	100	0
1b	Antenatal or postnatal women	100	0
1c	Patients with cancer	100	0
1d	Patients with heart disease	92	8
1e	Patients with bowel disorders (e.g. peptic ulcer, inflammatory bowel disease)	100	0
1f	Patients with renal disease	100	0
1g	Elderly populations	92	8
1h	Patients on extracorporeal membrane oxygenation (ECMO)	92	8
2	Other conditions that are prone to adverse effects of iron deficiency anaemia (IDA):		
2a	Acute brain injury	46*	54
2b	Septic shock	54*	46
1.2. Preoperative screening for IDA			
3	For surgical populations, the optimal preoperative haemoglobin (Hb) levels should be ≥ 13.0 g/dL in males and ≥ 12.0 g/dL in females	62*	31
4	All surgical populations should be routinely screened for anaemia, focusing on absolute or functional iron deficiency	85	15
5	Preoperative screening for IDA facilitates diagnosis and earlier administration of oral or intravenous iron therapy, thereby reducing hospitalisations for blood transfusions and contributing to higher rates of same-day surgery	85	15
6	It may be worthwhile to adopt preoperative protocol-driven evaluation for IDA	100	0
7	Hospital- or department-specific algorithms should be established to govern preoperative screening and management of IDA, including the roles of nurses, anaesthesiologists, physicians, haematopathologists, and surgeons	100	0
8	A nurse-led clinic can be implemented to facilitate preoperative screening for IDA, with the roles including:		
8a	Interpretation of protocol-driven anaemia evaluation	92	8
8b	Patient education on iron deficiency treatments, including effectiveness and tolerability of oral and intravenous iron therapies	100	0
8c	Administration of intravenous iron therapy when necessary, using dosing tools	69*	31
8d	Alerting attending surgeons and anaesthesiologists to unexplained or severe anaemia cases, and to consider referral to physicians for further work-up	100	0
9	Clinical management systems should be enhanced to track patient outcomes and automatically notify attending surgeons and anaesthesiologists if rechecked Hb remains below the target within 7–10 days before surgery	77*	23
10	Further local research should be conducted to assess the prevalence of anaemia and their causes among surgical populations in Hong Kong	100	0
1.3. Preoperative measures to optimise Hb levels in the general population			
*1.3.1. Patients undergoing elective surgery*			
11	In patients with suboptimal preoperative Hb levels, delay of elective surgery should be considered, and an iron supplement should be administered to correct the deficiency while investigating the underlying causes	69*	23
12	Transfusions should be avoided in patients undergoing elective surgery when their IDA is clinically stable and correctable	77*	23
*1.3.2. Patients undergoing urgent surgery*			
13	In patients who require more urgent surgery (e.g. within 2–4 weeks), preoperative IDA should be treated with intravenous iron therapy	77*	23
14	To treat preoperative anaemia in patients who are clinically unstable or require urgent surgery, transfusions (preferably with a restrictive Hb threshold and a single-unit approach [refer to Part 3]) may be considered	85	8
1.4. Preoperative measures to optimise Hb levels in special populations			
*1.4.1. Gynaecological patients*			
15	In patients with heavy menstrual bleeding and severe anaemia (i.e. Hb < 7 g/dL) due to fibroids:		
15a	Preoperative use of gonadotropin-releasing hormone (GnRH) agonists for 3–4 months can be considered to reduce intraoperative blood loss	54*	46
15b	Intravenous iron therapy ± a single-unit transfusion can be considered to optimise preoperative Hb levels, provided the patient is haemodynamically stable	85	15
15c	Early surgery should be considered in those who have already planned for the procedure	85	15
*1.4.2. Obstetric patients*			
16	In antenatal patients, screening of serum ferritin levels can be considered for early detection of IDA	100	0
17	The following treatment measures can be considered in antenatal patients:		
17a	Iron-rich diet	85	15
17b	Oral iron supplementation	92	8
17c	Intravenous iron therapy in the 2nd or 3rd trimesters (especially when oral iron therapy is contraindicated, intolerable, or ineffective)	100	0
18	Regarding the administration of intravenous iron therapy, the need for monitoring for any potential adverse reactions under an institutional protocol should be discussed with parturients in advance	85	15
*1.4.3. Renal patients*			
19	Higher thresholds (i.e. serum ferritin ≤ 800 ng/mL and transferrin saturation < 30%) would increase the sensitivity of identifying IDA in patients with chronic kidney disease	77*	23
20	To treat anaemia in patients with chronic kidney disease:		
20a	Intravenous iron therapy is preferred over oral iron therapy, especially when response to oral iron therapy is suboptimal	38*	62
20b	Erythropoiesis-stimulating agents can be considered in selected cases	100	0
20c	A conservative Hb target should be maintained	85	15
20d	Coordination with nephrologists should be considered when necessary	100	0
*1.4.4. Patients with bowel disorders*			
21	Screening for haematinics deficiency, including iron profile, vitamin B12 and folate, is recommended	85	8
22	Intravenous iron therapy, instead of oral iron therapy, is recommended in patients with iron deficiency due to malabsorption by the gastrointestinal tract	85	15
*1.4.5. Elderly patients*			
23	Routine screening for medications that pose a high risk for anaemia is recommended	85	8
24	Intravenous iron therapy may be preferred over oral iron therapy in patients with polypharmacy	69*	31
1.5. Patient empowerment			
25	Public education in the primary and community healthcare settings should be enhanced to raise awareness of IDA, especially among women at reproductive age	100	0
26	Patients should always be counselled regarding risks associated with anaemia and blood transfusions	100	0
27	Patient preferences, acceptance, or rejection regarding blood components and/or blood conservation modalities should be discussed preoperatively	100	0
28	Related consent forms and advanced directives should be obtained and documented preoperatively to ensure that acceptable options for optimal care are provided	92	8
**Part 2. Minimising perioperative blood loss**			
2.1. Surgeries and conditions associated with substantial blood loss			
1	Perioperative bleeding is common in diverse surgical fields, including:		
1a	Trauma surgery	100	0
1b	Orthopaedic surgery	100	0
1c	Neurosurgery	62*	38
1d	Visceral and transplant surgery	92	8
1e	Cardiac and vascular surgery	100	0
1f	Obstetric and gynaecological surgery	100	0
2	In the preoperative phase, interventions should be initiated to identify patients who may be at higher risk for bleeding, including those with:		
2a	Underlying coagulation abnormalities (inherited or acquired)	100	0
2b	Antithrombotic medications	100	0
2.2. Measures to minimise intraoperative blood loss in the general population			
*2.2.1. Surgical techniques*			
3	Judicious use of minimally invasive surgery, electrocautery, tourniquets, topical haemostatic agents (including mechanical and active biologic agents), and intraoperative blood salvage should be considered to reduce blood loss	85	15
*2.2.2. Patient positioning*			
4	Correct patient positioning is a simple and effective intervention to minimise intraoperative blood loss	92	8
5	The general principles of patient positioning include elevation of surgical sites and slow transition of positions	92	8
*2.2.3. Normothermia*			
6	Maintaining perioperative normothermia is crucial to reduce blood loss and the need for blood transfusions	100	0
*2.2.4. Point-of-care (POC) coagulation testing*			
7	POC testing of blood coagulation using a viscoelastic haemostatic assay (e.g. rotational thromboelastometry or thromboelastography) or ultrasound-induced resonance helps reducing the requirement for blood product transfusions by guiding the haemostatic therapy	92	8
8	POC testing of blood coagulation is recommended in cases of suspected coagulopathy or surgical settings where massive haemorrhage is anticipated (e.g. trauma, cardiac, liver, obstetric and gynaecological surgery)	100	0
*2.2.5. Haemostatic agents*			
9	Tranexamic acid can be administered perioperatively to reduce the risk of major bleeding in non-cardiac surgeries	100	0
10	Fibrinogen concentrate helps minimising intraoperative blood loss in certain surgical populations, including cardiac surgery, massive obstetric haemorrhage, or polytrauma with severe bleeding	100	0
11	In patients with expected massive haemorrhage awaiting viscoelastic or laboratory tests, administering 2-g fibrinogen concentrate based on clinical criteria at admission—such as low systolic blood pressure, metabolic acidosis, or low Hb levels—helps to provide initial coagulation support and correct hypofibrinogenemia	77*	23
*2.2.6. Reversal agents for anticoagulation*			
12	Prothrombin complex concentrate (PCC) and vitamin K1 are indicated for the urgent reversal of anticoagulation in patients with major acute bleeding, such as intracerebral haemorrhage, or those requiring emergency surgery who are taking warfarin	85	15
13	Regarding the use of specific reversal agents:		
13a	Idarucizumab should be considered in patients taking dabigatran if urgent reversal of anticoagulation is indicated; for example, in major acute bleeding or before emergency surgery	85	15
13b	Andexanet alfa, the reversal agent for factor Xa inhibitors (e.g. rivaroxaban, apixaban, and edoxaban), is not readily available in Hong Kong. If rapid reversal of an oral factor Xa inhibitor is indicated, PCC could be considered	85	15
14	PCC may serve as a treatment option for acute massive haemorrhage (not warfarin-induced) in patients undergoing surgery	38*	54
15	In acute massive haemorrhage, the advantages of PCC over plasma may include faster onset of action, off-the-shelf availability, no thawing requirement, and reduced risk of fluid overload	92	8
16	The limitations of PCC in the management of acute massive haemorrhage include the inability to replenish all clotting factors and the thrombotic risk associated with an overdose. Its use is preferably guided by POC coagulation testing	85	15
*2.2.7. Advanced haemodynamic monitoring*			
17	In surgical populations at high risk for massive bleeding, advanced haemodynamic monitoring providing continuous data (e.g. cardiac output, fluid responsiveness) can be considered to facilitate targeted and timely interventions, thereby minimising intraoperative blood loss and the need for blood transfusions, and improving patient outcomes	92	8
2.3. Measures to minimise intraoperative blood loss in special populations			
*2.3.1. Orthopaedic patients*			
18	During prone spine surgery, the abdomen should be well positioned to avoid compressing the inferior vena cava	100	0
19	Closed suction drains are not recommended in hip and knee arthroplasty	69*	31
*2.3.2. Obstetric patients*			
20	Prevention of postpartum haemorrhage (PPH; defined as a blood loss of ≥ 500 mL within 24 h after vaginal or caesarean delivery) is important in obstetric populations	100	0
21	To reduce the risk of PPH, the umbilical cord can be managed as follows:		
21a	For vaginal delivery, controlled cord traction can be offered routinely during the 3rd stage of labour, provided that the birth attendant has the necessary skills	100	0
21b	For caesarean delivery, controlled cord traction is recommended for the removal of the placenta	100	0
21c	Delayed (instead of early) cord clamping is recommended for all births unless the neonate is asphyxiated and needs to be moved immediately for resuscitation	100	0
22	The use of oxytocin or carbetocin to prevent PPH during the 3rd stage of labour is recommended for all births:		
22a	Carbetocin is recommended in women undergoing caesarean delivery, and for those undergoing vaginal delivery who are at increased risk for PPH	100	0
22b	Oxytocin is recommended in women undergoing vaginal delivery who do not have risk factors for PPH	85	15
23	The use of prophylactic tranexamic acid is recommended for high-risk patients with PPH	100	0
24	Second-line measures, including uterine compression sutures, balloon tamponade, and uterine artery embolisation, should be implemented early and with a lower threshold to help preventing PPH	100	0
25	Recommended treatments for PPH include:		
25a	Uterine massage	100	0
25b	Intravenous oxytocin alone as the first-line uterotonic treatment	69*	31
25c	Second-line uterotonics (e.g. syntometrine, carboprost, misoprostol) if bleeding does not respond to oxytocin	92	8
25d	Intravenous tranexamic acid administered as soon as possible (within 3 h) after bleeding onset, in addition to standard care	100	0
25e	Uterine balloon tamponade as a non-surgical treatment approach for PPH due to uterine atony if uterotonics are ineffective or unavailable	100	0
25f	Surgical intervention (e.g. compression suture, uterine and internal iliac artery ligation, hysterectomy) if PPH does not respond to uterotonics or other conservative treatments	100	0
25g	Single-unit transfusions may be considered	46*	38
25h	Iron repletion may be considered	77*	8
26	Placenta accreta spectrum, often related to prior caesarean sections, is a major risk factor for massive PPH	100	0
27	In patients with placenta accreta spectrum following caesarean sections, conservative management involving retention of the placenta in situ may be considered to minimise total blood loss, although the possibility of requiring hysterectomy cannot be ruled out	92	8
*2.3.3. Patients on antithrombotic therapy*			
28	In patients on antithrombotic therapy (e.g. warfarin, direct oral anticoagulants, antiplatelets) who plan to undergo elective surgery, the decision to stop antithrombotic therapy should consider the following factors:		
28a	Thrombotic risk associated with anticoagulation interruption in the perioperative period	100	
28b	Surgery/procedure-related bleeding risk	92	8
29	Antithrombotic therapy can be continued in surgeries/procedures with minimal bleeding risk, whereas heparin bridging may be needed in patients taking warfarin and at high risk for thromboembolism during surgeries/procedures with bleeding risk (e.g. recent venous thromboembolism and mechanical heart valves)	69*	23
30	Considering the wide variation in surgical procedures and patient comorbidities, thrombotic and bleeding risk assessment should be individualised and managed through a multidisciplinary approach	100	0
**Part 3. Rationalising the use of blood and blood components**			
3.1. Transfusion triggers			
1	In most surgical populations, there is no significant difference in clinical outcomes between restrictive and liberal transfusion strategies; however, restrictive strategies (i.e. lower Hb threshold and fewer transfusions of red blood cells) aim to minimise unnecessary blood use and optimise patient safety by reducing transfusion risk	77*	23
2	A restrictive transfusion threshold of Hb 7–8 g/dL is recommended for most surgical populations, including those undergoing non-cardiac surgery and those with critical conditions (e.g. patients admitted to the intensive care unit)	62*	38
3	A transfusion threshold of Hb 8 g/dL is recommended for patients with stable cardiovascular disease. Consider a more liberal threshold (Hb 9–10 g/dL) for acute coronary syndrome or anaemic heart failure	92	0
4	Transfusion thresholds should be individualised in patients receiving ECMO (venovenous-ECMO target Hb 7 g/dL [mainly for respiratory failure]; venoarterial-ECMO target Hb 8–9 g/dL [mainly for circulatory failure]), accounting for individual clinical status and context	69*	31
5	When indicated, single-unit transfusions with reassessment should replace multiunit orders	100	0
6	Transfusion strategies should be adjusted based on patient-specific factors, including laboratory data, clinical context, symptoms, and signs (Table 2), in addition to Hb levels	92	8
7	Routine use of physiologic transfusion triggers (e.g. systemic oxygen delivery, ST segment changes on electrocardiogram, mixed venous oxygen saturation, lactate levels, near-infrared spectroscopy) is not recommended because of a lack of high-level evidence	54*	46
3.2. Implementation of transfusion protocols			
8	Suggested key components of a hospital-wide transfusion protocol include:		
8a	Clear Hb thresholds tailored to patient subgroups	85	15
8b	Mandatory Hb check before transfusions when clinical context allows, and with application of POC coagulation testing as necessary	62*	38
8c	Assessment of coagulation, biochemical and metabolic parameters	69*	23
8d	Mandatory assessment of patient conditions before transfusion	100	0
8e	Single-unit transfusion orders as far as possible	85	15
8f	Checklists to ensure reassessments after single-unit transfusions	85	15
8g	Monitoring for adverse effects (e.g. ischaemic complications, haemodynamic instability, organ dysfunction)	92	8
9	Suggested measures to facilitate the implementation of transfusion strategies include:		
9a	Staff training on transfusion indications, restrictive thresholds and the single-unit policy	92	8
9b	Electronic reminders to confirm the need for transfusion in patients with Hb 7–8 g/dL without symptoms	54*	38
9c	Transfusion audits to monitor compliance, transfusion reactions, and clinical outcomes	92	8
9d	Feedback and refreshment training for clinicians based on audit data	100	0
9e	Refinement of protocols based on audit data	85	15
3.3. Advantages and challenges of patient blood management (PBM) programmes			
10	PBM programmes consistently deliver healthcare savings via improvements in patient outcomes, including shortening hospital stays, reducing postoperative complications, and minimising unnecessary blood transfusions	92	8
11	PBM programmes enhance resource sustainability and ethical use of blood products	92	8
12	Disease-specific and specialty-specific protocols are needed to consistently implement PBM programmes	77*	23
13	Key challenges and barriers towards the implementation of PBM include hospital culture, inadequate staff awareness, poor interdisciplinary communication or collaboration, absence of electronic monitoring systems, and resource limitations (staff, time, and finances)	77*	23

^*^Statements with < 80% of panellists choosing ‘accept completely’

## Part 1. Optimising patients’ RBC mass and improving anaemia management

### Groups at high risk for iron deficiency and anaemia

Statement 1. Groups at high risk for iron deficiency and anaemia include (Table 2) the following:

- Women at reproductive age.
- Antenatal or postnatal women.
- Patients with cancer.
- Patients with heart disease.
- Patients with bowel disorders (e.g. peptic ulcer, inflammatory bowel disease).
- Patients with renal disease.
- Elderly populations.
- Patients on extracorporeal membrane oxygenation (ECMO).

**Table 2:** High-risk groups and corresponding suggested haemoglobin (Hb) thresholds for transfusion

Illness or age group		Potential causes and related adverse effects	Suggested transfusion Hb threshold*	Threshold adjustment criteria
Groups at high risk for iron deficiency and anaemia	Women of reproductive age	Heavy menstrual bleeding from various causes, e.g. dysfunctional uterine bleeding, uterine fibroids, adenomyosis	7–8 g/dL	Symptoms of anaemia (e.g. angina, dyspnoea)
	Antenatal or postnatal women	Iron, folate, or vitamin B12 deficiency Postpartum haemorrhage	7–8 g/dL	Symptoms of anaemia (e.g. angina, dyspnoea)
	Cancer	Cancer bleeding Chronic inflammation	7–8 g/dL	Symptoms of anaemia (e.g. angina, dyspnoea)
	Heart disease	Nutritional deficiency GI blood loss associated with antiplatelet agents Functional deficiency due to chronic inflammation	Stable CVD: 8 g/dL ACS or anaemic HF: 9–10 g/dL	Active ischaemia Haemodynamic instability Ongoing myocardial infarction Cardiogenic shock
	Bowel disorders (e.g. peptic ulcer, IBD)	Malabsorption of haematinics GI bleeding GI or systemic inflammation	7–8 g/dL	7 g/dL if GI bleeding
	Renal disease	Inflammation Malabsorption of iron	7–8 g/dL	Symptoms of anaemia (e.g. angina, dyspnoea)
	Elderly populations	Nutritional deficiencies Chronic inflammation Chronic kidney disease Polypharmacy (e.g. PPIs, anticoagulants, aspirin)	7–8 g/dL	Liberalise threshold slightly (e.g. Hb 8 g/dL) if poor physiologic reserve
	Patients on ECMO	Frequent blood draws Anticoagulation Haemolysis	VV-ECMO: 7 g/dL VA-ECMO: 8–9 g/dL	Cardiotomy
Groups at high risk for adverse effects of IDA	Acute brain injury	Extracranial traumas	Individualised (7–9 g/dL)	Signs of cerebral hypoxia
	Septic shock	Inflammation Haemolysis	7 g/dL	Persistent lactic acidosis Poor perfusion

^*^In patients with chronic anaemia, avoid transfusions unless symptomatic (adapt to baseline Hb)

*ACS* acute coronary syndrome, *CVD* cardiovascular disease, *ECMO* extracorporeal membrane oxygenation, *GI* gastrointestinal, *HF* heart failure, *IBD* inflammatory bowel disease, *IDA* iron deficiency anaemia, *PPI* proton pump inhibitor, *VA* venoarterial, *VV* venovenous

A recent public health survey conducted by the Hong Kong Department of Health found that iron deficiency and IDA are common among local females at reproductive age (18–49 years), with a prevalence of 17.5% and 10.6%, respectively, compared with 2.7% and 2.1% in postmenopausal women [7].

Antenatal and postnatal women are also commonly affected by iron deficiency and IDA. Data from Australia, Canada, and the USA showed that 40–70% of pregnant women experience iron deficiency [8]. The WHO estimates that 37% of pregnant women worldwide experience anaemia, which is primarily caused by iron deficiency, with the WHO region of Southeast Asia exhibiting a higher burden [9]. In China, anaemia, iron deficiency, and IDA are estimated to affect 30.7%, 45.6%, and 17.3% of pregnant women, respectively [10].

Anaemia is prevalent among cancer patients and often arises from multiple factors [11–13]. Common causes include absolute iron deficiency due to bleeding (especially in patients with colorectal, urological, and gynaecological malignancies); chronic inflammation causing iron sequestration due to increased hepcidin levels; and treatment-related myelosuppression.

Iron deficiency and anaemia occur in 40–60% and 30–50% of patients with heart failure, respectively [14, 15]. Common causes of these closely related conditions include nutritional deficiency, antiplatelet-led gastrointestinal bleeding, functional deficiency due to chronic inflammation, and fluid retention-associated dilutional anaemia [14, 16]. However, iron deficiency is often unrecognised and undertested in patients with heart failure [17]. Correction of iron deficiency may reduce readmissions for heart failure [18].

Anaemia affects up to 60% of patients with non-malignant gastrointestinal diseases, including peptic ulcer, inflammatory bowel disease, and atrophic gastritis [19]. These conditions frequently involve gastrointestinal tract bleeding and systemic inflammation, impairing the absorption of haematinics (i.e. iron, vitamin B12, and folate), increasing the risk of absolute and functional iron deficiency and anaemia [19].

Chronic kidney disease (CKD) may raise the risk of anaemia through reduced erythropoietin levels and responsiveness, iron deficiency, chronic inflammation, and a decreased lifespan of RBCs [20]. A systematic review and meta-analysis of 86 studies across 10 Asian countries found that 12–57% of patients at different stages of CKD experience anaemia, with an overall prevalence of 42% [21].

Elderly populations are prone to iron deficiency and anaemia because of nutritional deficiencies (e.g. iron and vitamin B12); underlying conditions (e.g. CKD and chronic inflammation); and polypharmacy (e.g. proton pump inhibitors for bowel disorders, and anticoagulants and antiplatelets for cardiovascular disease) that may reduce iron absorption or increase the risk of bleeding [22, 23]. Anaemia is associated with increased risks of frailty, falls, cognitive decline, and mortality in elderly populations [24].

In patients receiving venovenous (VV) or venoarterial (VA) ECMO, anaemia is common and often caused by multiple factors, including circuit-related haemolysis, bleeding due to anticoagulation or platelet dysfunction, and frequent phlebotomy [25, 26]. These patients also frequently experience complications of anaemia, such as haemodynamic instability, coagulopathy, and multiorgan dysfunction [25, 26].

Statement 2. Other conditions that are prone to adverse effects of IDA:

- Acute brain injury.
- Septic shock.

In cases of acute brain injury, Hb levels play a crucial role in determining brain oxygenation, and anaemia is linked to poorer neurological outcomes and higher mortality rates [27]. However, it remains unclear whether anaemia directly impacts neurological recovery or only indicates a more severe disease that necessitates extended hospitalisation [27].

A prospective cohort study of 90 patients with sepsis showed that approximately half had iron deficiency and IDA, based on reticulocyte Hb equivalent and Hb concentration [28]. Anaemia is associated with longer hospital stays and a higher risk of mortality in patients with sepsis [29, 30]. Optimising iron concentrations is important to enhance immunity and aerobic metabolism, facilitating recovery from sepsis.

### Preoperative screening for IDA

- Statement 3. For surgical populations, the optimal preoperative Hb levels should be ≥ 13.0 g/dL in males and ≥ 12.0 g/dL in females.
- Statement 4. All surgical populations should be routinely screened for anaemia, focusing on absolute or functional iron deficiency.
- Statement 5. Preoperative screening for IDA facilitates diagnosis and earlier administration of oral or intravenous (IV) iron therapy, thereby reducing hospitalisations for blood transfusions and contributing to higher rates of same-day surgery.

The WHO defines anaemia as Hb < 13 g/dL for males and Hb < 12 g/dL for females [31]. The prevalence of preoperative anaemia, primarily due to absolute iron deficiency and iron sequestration, ranges from 30 to 40% among the general surgical population [2, 32]. Preoperative anaemia is associated with worsened clinical outcomes (e.g. increased rates of postoperative morbidity, mortality, and readmission) and higher risks of undergoing transfusion and exposure to related adverse effects [2, 32]. Therefore, all surgical populations should be screened for anaemia preoperatively, allowing for early initiation of appropriate treatment, such as oral or IV iron therapy in cases of iron deficiency, to improve clinical outcomes and avoid transfusions [2].

- Statement 6. It may be worthwhile to adopt preoperative protocol-driven evaluation for IDA.
- Statement 7. Hospital- or department-specific algorithms should be established to govern preoperative screening and management of IDA, including the roles of nurses, anaesthesiologists, physicians, haematopathologists, and surgeons.

In patients with preoperative anaemia, it may be worthwhile to check their iron profiles to identify the aetiology, unless clinically relevant conditions pre-exist. Okocha et al. suggested that preoperative anaemia evaluation be conducted by simultaneously collecting two blood samples from a patient; a complete blood count (CBC) is processed on the first sample, and an iron reflex test is performed on the second sample only if anaemia is confirmed [33]. Meanwhile, the panel considered that a hospital or department should adopt a specific algorithm for checking and managing preoperative IDA.

In general, a preoperative medicine clinic, possibly led by an anaesthesiologist, should be established to assess surgical patients for anaemia on a CBC at an early stage (i.e. before pathological diagnosis and functional capacity assessments). Additionally, there should be a protocol to refer patients diagnosed with IDA to iron replacement therapy (e.g. a nurse-led IV iron clinic).

Statement 8. A nurse-led clinic can be implemented to facilitate preoperative screening for IDA, with the roles including the following:

- Interpretation of protocol-driven anaemia evaluation
- Patient education on iron deficiency treatments, including effectiveness and tolerability of oral and IV iron therapies
- Administration of IV iron therapy when necessary, using dosing tools
- Alert attending surgeons and anaesthesiologists to unexplained or severe anaemia cases, and consider referral to physicians for further work-up

Nurses play a crucial role in optimising PBM by providing patient education and anaemia care services [34]. For patients with preoperative IDA who are scheduled for major elective surgeries, implementing a nurse-led IV iron protocol may help to reduce the incidence of perioperative blood transfusions and the associated risks [35]. Table 3 lists the proposed criteria for considering IV iron and other measures to address preoperative IDA.

**Table 3:** Proposed criteria for considering measures to address preoperative iron deficiency anaemia (IDA)

Step	Threshold	Action
Screen	Hb < 13.0 g/dL (male); or Hb < 12.0 g/dL (female)	Trigger reflex iron panel; plan optimisation
Diagnose IDA	Ferritin < 30 µg/L; or Ferritin 30–100 µg/L + TSAT < 20% ± ↑CRP	Consider surgery delay and start iron therapy: - If > 4 weeks before surgery, consider oral iron + vitamin C; recheck in 2–4 weeks - If intolerance/poor response to oral iron, consider IV iron - If ≤ 4 weeks before surgery, consider IV iron (single or split dose); recheck in ~ 2–3 weeks
Transfusion	Untreatable IDA; Unstable haemodynamics; or Urgent surgery	Consider restrictive, single-unit policy whenever applicable

*CRP* C-reactive protein, *Hb* haemoglobin, *IV* intravenous, *TSAT* transferrin saturation

Notably, even in the same hospital, different departments (e.g. surgery and anaesthesiology) may have separate protocols for preoperative anaemia management. To facilitate the implementation of an aligned protocol or algorithm across departments, it is important to have a clinician (e.g. anaesthesiologist) serve as a coordinator to inform colleagues about the potential impact (e.g. increased demand for personnel and resources due to more frequent phlebotomy) and benefits (e.g. reduced incidence of transfusion-led hospitalisations, enhanced rate of same-day surgery, and improved patient outcomes). Furthermore, a new algorithm can be initially piloted and evaluated in one or two departments; if the results are promising, it may then be rolled out to additional departments.

- Statement 9. Clinical management systems should be enhanced to track patient outcomes and automatically notify attending surgeons and anaesthesiologists if rechecked Hb remains below the target within 7–10 days before surgery.
- Statement 10. Further local research should be conducted to assess the prevalence of anaemia and their causes among surgical populations in Hong Kong.

With respect to future perspectives, the panel suggested that hospitals strengthen tracking of preoperative Hb levels by upgrading clinical management systems. They also highlighted that more research should be done to determine the prevalence of anaemia, its causes, and rates of related admissions among patients scheduled for elective surgeries in Hong Kong. These data may help to differentiate the characteristics of preoperative anaemia between Asian and Western surgical populations.

### Preoperative measures to optimise Hb levels in the general population

#### Patients undergoing elective surgery

- Statement 11. In patients with suboptimal preoperative Hb levels, delay of elective surgery should be considered, and an iron supplement should be administered to correct the deficiency while investigating the underlying causes.
- Statement 12. Transfusions should be avoided in patients undergoing elective surgery when their IDA is clinically stable and correctable.

Oral iron supplement (once-daily or alternate-day dosing, preferably plus vitamin C) is the first-line treatment for preoperative IDA in non-urgent surgeries (i.e. can be performed ≥ 4 weeks later) [36]. Tolerability and response to oral iron should be checked at 2–4 weeks after treatment initiation [36]. Any suboptimal finding prompts the use of IV iron [36]. If anaemia persists, non-iron causes (e.g. gastrointestinal bleeding, vitamin B12/folate deficiency, renal problem, thalassaemia) and referral to specialists should be considered [36]. The preoperative Hb level should be optimised to ≥ 13.0 g/dL for males and ≥ 12.0 g/dL for females (Sect. ‘Preoperative screening for IDA’, Statement 3), reducing the need for perioperative transfusions.

#### Patients undergoing urgent surgery

- Statement 13. In patients who require more urgent surgery (e.g. within 2–4 weeks), preoperative IDA should be treated with IV iron therapy.
- Statement 14. To treat preoperative anaemia in patients who are clinically unstable or require urgent surgery, transfusions (preferably with a restrictive Hb threshold and a single-unit approach (refer to Part 3)) may be considered.

IDA before urgent surgeries (i.e. will be performed < 4 weeks later) should be treated with IV iron (doses calculated using a simplified regimen or the Ganzoni formula), instead of oral iron [36]. Optimisation of the Hb level is expected to be achieved after 2–3 weeks of IV iron treatment [36]. Multiple studies demonstrated that IV iron is associated with increased Hb levels, as well as reductions in transfusion requirements and the risk of postoperative mortality among a wide range of surgical populations with preoperative IDA [37–41]. If surgical urgency or an unstable patient condition precludes iron treatment, cautious transfusions may be considered to rectify preoperative anaemia (Part 3) [36].

### Preoperative measures to optimise Hb levels in special populations

#### Gynaecological patients

Statement 15. In patients with heavy menstrual bleeding and severe anaemia (i.e. Hb < 7 g/dL) due to fibroids

- Preoperative use of gonadotropin-releasing hormone (GnRH) agonists for 3–4 months can be considered to reduce intraoperative blood loss.
- IV iron therapy ± a single-unit transfusion can be considered to optimise preoperative Hb levels, provided the patient is haemodynamically stable.
- Early surgery should be considered in those who have already planned for the procedure.

A systematic review and meta-analysis revealed that GnRH agonists effectively induce amenorrhea, diminish uterine volume, reduce blood loss, and raise Hb levels, facilitating avoidance of preoperative transfusions and IV iron use [42]. Protocol-driven IV iron use and transfusions can be considered to treat heavy menstrual bleeding. In addition to optimisation of Hb levels, prioritisation of early surgical intervention should also be considered to avoid repeated transfusions and IV iron use in patients with severe fibroids.

#### Obstetric patients

- Statement 16. In antenatal patients, screening of serum ferritin levels can be considered for early detection of IDA
- Statement 17. The following treatment measures can be considered in antenatal patients:
  - Iron-rich diet.
  - Oral iron supplementation.
  - IV iron therapy in the 2nd or 3rd trimester (especially when oral iron therapy is contraindicated, intolerable, or ineffective)
- Statement 18. Regarding the administration of IV iron therapy, the need for monitoring for any potential adverse reactions under an institutional protocol should be discussed with parturients in advance.

Ferritin should be screened in all pregnant women at the initial antenatal visit, at 24–28 weeks, and when a low mean corpuscular volume is noted [8, 43]. Patient education should be enhanced to highlight the importance of early antenatal care, including an iron-rich diet and oral iron supplementation, in preventing and treating IDA. Although contraindicated in the first trimester, IV iron is applicable during the second and third trimesters in pregnant women with low ferritin levels (i.e. < 30 ng/mL) and/or low Hb levels (i.e. < 9 or 10 g/dL) [44]. The considerations for using IV iron, particularly the need for adverse effects monitoring, should be discussed with parturients before treatment initiation.

#### Renal patients

- Statement 19. Higher thresholds (i.e. serum ferritin ≤ 800 ng/mL and transferrin saturation [TSAT] ≤ 30%) could increase the sensitivity of identifying IDA in patients with CKD.
- Statement 20. To treat anaemia in patients with CKD:
  - IV iron therapy is preferred over oral iron therapy, especially when response to oral iron therapy is suboptimal.
  - Erythropoiesis-stimulating agents (ESAs) can be considered in selected cases.
  - A conservative Hb target should be maintained.
  - Coordination with nephrologists should be considered when necessary.

The 2012 Kidney Disease: Improving Global Outcomes (KDIGO) guidelines recommend iron repletion for patients with CKD who have serum ferritin levels ≤ 500 ng/mL and a TSAT ≤ 30% [45]. However, other international guidelines suggest a ferritin ceiling of 800 ng/mL during iron supplementation because many patients with CKD have ferritin levels > 500 ng/mL [46]. Considering these recommendations, the panel agreed upon serum ferritin levels ≤ 800 ng/mL and a TSAT ≤ 30% as the thresholds for identifying IDA in patients with CKD.

IV iron is preferred over oral iron to treat anaemia in patients with CKD. Furthermore, the randomised PIVOTAL trial showed that, in patients undergoing haemodialysis, high-dose IV iron (400 mg monthly, unless ferritin > 700 ng/mL or TSAT ≥ 40%) significantly reduced the risk of the primary composite endpoint (nonfatal myocardial infarction, nonfatal stroke, hospitalisation for heart failure, or death; hazard ratio (HR), 0.85; 95% confidence interval (CI), 0.73–1.00; *P* = 0.04 for superiority) and lowered doses of ESAs used (median difference, −7539 IU; 95% CI, −9485 to −5582) compared with low-dose IV iron (0–400 mg monthly, administered only when ferritin < 200 ng/mL or TSAT < 20%) [47].

ESAs are feasible to treat anaemia in CKD; however, the cardiovascular risk linked to using these agents for normalising Hb levels remains a concern [36]. To optimise the efficacy of ESAs, concomitant iron supplementation should be considered to correct absolute or functional iron deficiency [36]. ESAs may be considered to treat preoperative anaemia in CKD patients who decline transfusions or have complex red cell antibodies [36]. The target Hb level for CKD patients receiving iron and ESA therapy ranges from 10 to 12 g/dL [36]. Referral to nephrologists for further management should be considered when necessary.

#### Patients with bowel disorders

- Statement 21. Screening for haematinics deficiency, including iron profile, vitamin B12, and folate, is recommended.
- Statement 22. IV iron therapy, instead of oral iron therapy, is recommended in patients with iron deficiency due to malabsorption by the gastrointestinal tract.

In patients with gastrointestinal disease, inflammation and haemorrhage are common causes of malabsorption and increased loss or utilisation of haematinics, primarily iron, vitamin B12, and folate, which often manifest clinically as anaemia [48]. The limitations of oral iron therapy include poor absorption and intolerance, particularly gastrointestinal side effects of nausea, bloating, and diarrhoea [49]. The preferred treatment for anaemia in patients with bowel disorders is IV iron, which offers a promising safety profile and a 65–75% response rate within 4–8 weeks [49].

#### Elderly patients

- Statement 23. Routine screening for medications that pose a high risk for anaemia is recommended.
- Statement 24. IV iron therapy may be preferred over oral iron therapy in patients with polypharmacy.

Elderly patients are prone to multimorbidity and subsequent polypharmacy. Many medications can raise the risk of IDA by influencing iron balance in the gastrointestinal tract, with potential mechanisms including impairment of haemostasis due to increased chronic blood loss (e.g. anticoagulants) or mucosal damage (e.g. non-steroidal anti-inflammatory drugs), and impairment of iron absorption (e.g. proton pump inhibitors) [50]. IV iron may offer faster and more effective treatment for IDA in elderly patients, particularly those with chronic conditions and polypharmacy, compared with oral iron, whose efficacy may be limited by suboptimal tolerance, absorption, or adherence [51, 52].

### Patient empowerment

- Statement 25. Public education in the primary and community healthcare settings should be enhanced to raise awareness of IDA, especially among women at reproductive age.
- Statement 26. Patients should always be counselled regarding risks associated with anaemia and blood transfusions.
- Statement 27. Patient preferences, acceptance, or rejection regarding blood components and/or blood conservation modalities should be discussed preoperatively.
- Statement 28. Related consent forms and advanced directives should be obtained and documented preoperatively to ensure that acceptable options for optimal care are provided.

Primary health education about the prevention and management of IDA, particularly the importance of iron-rich diet and adherence to oral iron supplementation, should be strengthened in a range of community settings, including women’s health clinics, elderly centres, school health programmes, and religious groups.

In preoperative anaemia management, educating patients on the risks related to anaemia and transfusions, as well as measures to mitigate these risks, is a pivotal part of informed consent, patient engagement, and shared decision-making, facilitating patient empowerment, treatment compliance, and recovery [53]. The role of patients should be shifted from passive recipients to active care partners who advocate for their own blood management and improved clinical outcomes [54]. It is crucial to establish clear two-way communication that allows for a transparent and understandable dialogue between the patient and healthcare provider [54].

As patient empowerment advances, PBM is expected to become increasingly personalised to address individual characteristics, preferences, and values in optimising red cell mass, minimising blood loss, and improving tolerance to anaemia [54]. The strategy may further evolve into a broader public health initiative, with increased engagement of patients and communities [54].

## Part 2. Minimising perioperative blood loss

### Surgeries and conditions associated with substantial blood loss

Statement 1. Perioperative bleeding is common in diverse surgical fields, including the following:

- Trauma surgery
- Orthopaedic surgery
- Neurosurgery
- Visceral and transplant surgery
- Cardiac and vascular surgery
- Obstetric and gynaecological surgery

Statement 2. In the preoperative phase, interventions should be initiated to identify patients who may be at higher risk for bleeding, including those with the following:

- Underlying coagulation abnormalities (inherited or acquired).
- Antithrombotic medications.

Perioperative bleeding is common among different surgical populations and patients with comorbidities, particularly coagulation disorders and cardiovascular conditions that require anticoagulants and/or antiplatelets. Preoperative checking for risk factors for bleeding is important. The management of perioperative bleeding requires a multimodal and multidisciplinary approach.

### Measures to minimise intraoperative blood loss in the general population

#### Surgical techniques

- Statement 3. Judicious use of minimally invasive surgery, electrocautery, tourniquets, topical haemostatic agents (including mechanical and active biologic agents), and intraoperative blood salvage should be considered to reduce blood loss.

Consistent with the panel’s consensus in 2020 [4], surgical techniques and topical haemostatic agents remain important to optimise haemostasis and minimise intraoperative blood loss. Additionally, blood (or cell) salvage is widely endorsed as a key perioperative PBM strategy. In a meta-analysis of 82 randomised trials with > 12,500 participants across a variety of surgical types, cell salvage reduced the risk of allogeneic transfusion (risk ratio (RR), 0.65; 95% CI, 0.59–0.72) compared with no cell salvage, without evidence of differences in perioperative adverse events [55]. Other studies showed that the quality of cell-salvaged RBCs is promising, with typically low concentrations of residual heparin [56]. Cell salvage is feasible in oncologic, obstetric, and contaminated surgeries, with risk reduction measures including appropriate washing, leukocyte depletion filters, and perioperative antibiotics [56]. Cell salvage is generally a cost-effective intervention, particularly when employed selectively for patients at high risk for transfusions [56].

#### Patient positioning

- Statement 4. Correct patient positioning is a simple and effective intervention to minimise intraoperative blood loss.
- Statement 5. The general principles of patient positioning include elevation of surgical sites and slow transition of positions.

The panel’s previous consensus highlighted these principles [4]. In general, positioning the operative site above heart level can reduce venous pressure and blood loss. Using the reverse Trendelenburg position is crucial for minimising blood loss during upper abdominal, neck, and head surgeries. Changing positions slowly can avoid sudden shifts in blood pressure and lower the risk of bleeding. Specific positioning should be considered to reduce blood loss in certain surgical procedures, particularly orthopaedic surgeries (refer to Sect. ‘Orthopaedic patients’).

#### Normothermia

- Statement 6. Maintaining perioperative normothermia is crucial to reduce blood loss and the need for blood transfusions.

Hypothermia impairs platelet function by diminishing the release of thromboxane A2, thereby contributing to coagulopathy and increased haemorrhage during surgical procedures. In a meta-analysis of randomised trials that included normothermic and mildly hypothermic surgical patients (median temperature difference, 0.85 °C), hypothermia significantly increased blood loss by ~16% (*P* = 0.009) and the RR for transfusion requirement by ~22% (*P* = 0.027), highlighting the importance of maintaining perioperative normothermia [57].

#### POC coagulation testing

- Statement 7. POC testing of blood coagulation using a viscoelastic haemostatic assay (VHA; e.g. rotational thromboelastometry (ROTEM) or thromboelastography (TEG)) or ultrasound-induced resonance helps reducing the requirement for blood product transfusions by guiding the haemostatic therapy.
- Statement 8. POC testing of blood coagulation is recommended in cases of suspected coagulopathy or surgical settings where massive haemorrhage is anticipated (e.g. trauma, cardiac, liver, obstetric and gynaecological surgery)

Emphasising the use of POC testing for blood coagulation represents a key update from the previous consensus. VHAs, such as ROTEM and TEG, and ultrasound-induced resonance are increasingly accepted POC bedside techniques to monitor real-time haemostatic competence and coagulation status in patients with massive haemorrhage or suspected coagulopathy [58, 59]. These tools facilitate rapid and sensitive phenotyping of haemostasis and identification of coagulation deficits, allowing for more individualised and timelier administration of blood components and haemostatic adjuncts to correct aberrant haemostasis [58, 59]. Employing VHA-guided trauma resuscitation and massive transfusions may help to reduce unnecessary blood transfusions and improve patient outcomes, including mortality, in various surgical populations at high risk of substantial blood loss [58, 59].

#### Haemostatic agents

- Statement 9. TXA can be administered perioperatively to reduce the risk of major bleeding in non-cardiac surgeries.

As suggested in the previous consensus, TXA is an important haemostatic agent that helps to reduce perioperative blood loss in trauma, orthopaedic, and other non-cardiac surgeries [4, 60]. Recently, the randomised placebo-controlled POISE-3 trial showed that, among 9535 patients undergoing non-cardiac surgeries, systemic administration of TXA (IV bolus, 1 g) significantly reduced the incidence of composite bleeding outcomes by 24% (*P* < 0.001), with consistent effects across surgical types and no increased risk of thrombotic events; however, the noninferiority of TXA in terms of composite cardiovascular outcomes (14.2% vs. 13.9% for placebo; HR, 1.02; 95% CI, 0.92–1.14; *P* = 0.04) was not established [61]. Previously, a randomised trial of 4631 patients undergoing coronary-artery surgery demonstrated that, compared with placebo, TXA significantly reduced total units of transfusions (4331 vs. 7994; *P* < 0.001) and the incidence of major bleeding or cardiac tamponade leading to reoperation (1.4% vs. 2.8%; *P* = 0.001). However, TXA did not significantly improve the primary composite outcome of death and thrombotic complications within 30 days post-surgery (16.7% vs. 18.1%; RR, 0.92; 95% CI, 0.81–1.05; *P* = 0.22), and was significantly associated with a higher risk of postoperative seizures (0.7% vs. 0.1%; *P* = 0.002) [62]. Therefore, the use of TXA in cardiac surgery should be cautious.

- Statement 10. Fibrinogen concentrate helps minimising intraoperative blood loss in certain surgical populations, including cardiac surgery, massive obstetric haemorrhage, or polytrauma with severe bleeding.
- Statement 11. In patients with expected massive haemorrhage awaiting viscoelastic or laboratory tests, administering 2-g fibrinogen concentrate based on clinical criteria at admission—such as low systolic blood pressure, metabolic acidosis, or low Hb levels—helps to provide initial coagulation support and correct hypofibrinogenemia.

Highlighting the utility of fibrinogen concentrate is an update from the previous consensus. There is an upward trend in its adoption to control intraoperative blood loss. Studies showed that fibrinogen concentrate reduced blood loss in patients undergoing cardiac surgery or experiencing massive obstetric haemorrhage or polytrauma along with hypofibrinogenaemia [63–65]. A meta-analysis of 13 randomised placebo-controlled trials with 900 surgical patients revealed that fibrinogen concentrate significantly reduced postoperative blood loss in the first 12 h by a mean of 134.6 mL and increased clot firmness in thromboelastometry by a mean of 2.5 mm, with no increased incidences of thromboembolism, myocardial infarction, or acute kidney injury [66]. A recent phase 3 randomised trial of 222 patients undergoing major spinal surgery or cytoreductive surgery for pseudomyxoma peritonei demonstrated that fibrinogen concentrate was associated with a lower volume of intraoperative blood loss (1381 vs. 1660 mL; least squares mean difference, −279 mL vs. a non-inferiority margin of 150 mL; *P* < 0.0001) and lower incidences of serious adverse events (25% vs. 37%) and thromboembolic events (4% vs. 12%) compared with fresh frozen plasma or cryoprecipitate [67]. Notably, a task force of experts in bleeding care from several European countries [68] has recommended that, in patients with expected massive bleeding and certain clinical signs at admission, i.e. systolic blood pressure < 100 mmHg, lactate ≥ 5 mmol/L, base excess ≤ −6, or Hb ≤ 9 g/dL, 2-g fibrinogen concentrate, instead of plasma transfusion, can serve as initial coagulation support to correct hypofibrinogenaemia, before viscoelastic or laboratory tests are conducted.

#### Reversal agents for anticoagulation

- Statement 12. Prothrombin complex concentrate (PCC) and vitamin K1 are indicated for the urgent reversal of anticoagulation in patients with major acute bleeding, such as intracerebral haemorrhage, or those requiring emergency surgery who are taking warfarin.
- Statement 13. Regarding the use of specific reversal agents:
  a Idarucizumab should be considered in patients taking dabigatran if urgent reversal of anticoagulation is indicated; for example, in major acute bleeding or before emergency surgery.
  b Andexanet alfa, the reversal agent for factor Xa inhibitors (e.g. rivaroxaban, apixaban, and edoxaban), is not readily available in Hong Kong. If rapid reversal of an oral factor Xa inhibitor is indicated, PCC could be considered.

The role of PCC (typically containing factors II, VII, IX, and X) and other reversal agents for anticoagulation in PBM is an emerging area of interest. PCC is indicated for reversing the anticoagulant effect of warfarin by replenishing vitamin K-dependent coagulation factors in patients with major bleeding or undergoing emergency surgery [69–71]. Vitamin K1 should be co-administered with PCC to sustain the reversal effect on warfarin, which has a long half-life [71]. To reverse the anticoagulant effects of direct oral anticoagulants (DOACs) in an emergency, specific antidotes, such as idarucizumab (for dabigatran) and andexanet alfa (for factor Xa inhibitors), should be used instead of PCC, when these agents are accessible [72–74].

- Statement 14. PCC may serve as a treatment option for acute massive haemorrhage (not warfarin-induced) in patients undergoing surgery.
- Statement 15. In acute massive haemorrhage, the advantages of PCC over plasma may include faster onset of action, off-the-shelf availability, no thawing requirement, and reduced risk of fluid overload.
- Statement 16. The limitations of PCC in the management of acute massive haemorrhage include the inability to replenish all clotting factors and the thrombotic risk associated with an overdose. Its use is preferably guided by POC coagulation testing.

PCC has been shown to be effective in reducing perioperative bleeding and transfusion requirements and improving the international normalised ratio (INR) in patients with and without coagulopathy [71, 75, 76]; however, these indications have not been approved by health authorities. The panellists shared that, in clinical practice, PCC has been used to treat intraoperative non-anticoagulated massive haemorrhage, attributable to its ability to rapidly replenish certain coagulation factors and reduce plasma use. They also suggested several potential benefits of PCC over plasma (Statement 15). However, the lack of some clotting factors in PCC may make it insufficient as a standalone treatment in severe trauma cases [76]. Another concern is the potential thrombotic risk of PCC [77]. To enhance the effectiveness and safety of PCC in treating intraoperative non-anticoagulated massive haemorrhage, its use is preferably guided by POC VHAs, which are important for monitoring coagulation status, mitigating the risk of prothrombotic complications, and helping to determine whether additional measures, such as fibrinogen or platelet transfusions, are necessary to further optimise the haemostatic balance [78]. Future studies are warranted to identify optimal dosing and timing of PCC for the treatment of non-anticoagulated massive haemorrhage among different surgical populations.

#### Advanced haemodynamic monitoring

- Statement 17. In surgical populations at high risk for massive bleeding, advanced haemodynamic monitoring providing continuous data (e.g. cardiac output, fluid responsiveness) can be considered to facilitate targeted and timely interventions, thereby minimising intraoperative blood loss and the need for blood transfusions, and improving patient outcomes.

Advanced haemodynamic monitoring provides real-time data on a patient’s blood flow and oxygen delivery [79]. By continuously tracking key variables such as cardiac output, clinicians can pre-emptively adjust fluid administration, vasopressors, and inotropic agents to help patients maintain stable haemodynamics, prevent perfusion-related complications, and reduce the likelihood of excessive bleeding and transfusions [79].

### Measures to minimise intraoperative blood loss in special populations

#### Orthopaedic patients

- Statement 18. During prone spine surgery, the abdomen should be well positioned to avoid compressing the inferior vena cava.
- Statement 19. Closed suction drains are not recommended in hip and knee arthroplasty.

Because the inferior vena cava (IVC) communicates with the epidural veins through a valveless system, any compression of the IVC can lead to engorgement of the epidural veins. To mitigate the risk of excessive bleeding from these engorged veins during prone spine surgery, compression should be avoided by positioning the abdomen appropriately using proper bolsters [80–82].

In hip and knee arthroplasty, the use of closed suction drains has been shown to correlate with an increased need for transfusions, and offers no significant benefits in terms of lowering the incidence of wound haematomas or preventing infection [83, 84].

#### Obstetric patients

- Statement 20. Prevention of PPH (defined as a blood loss of ≥ 500 mL within 24 hours after vaginal or caesarean delivery) is important in obstetric populations.
- Statement 21. To reduce the risk of PPH, the umbilical cord can be managed as follows:
  - For vaginal delivery, controlled cord traction (CCT) can be offered routinely during the 3rd stage of labour, provided that the birth attendant has the necessary skills.
  - For caesarean delivery, CCT is recommended for the removal of the placenta.
  - Delayed (instead of early) cord clamping is recommended for all births unless the neonate is asphyxiated and needs to be moved immediately for resuscitation.

A high incidence (~ 15% among obstetric patients across public hospitals in Hong Kong; ~30% among those undergoing caesarean delivery) and a significant correlation to maternal morbidity and mortality highlight the importance of preventing PPH [85–87].

A meta-analysis of randomised trials that compared the use and non-use of planned CCT in women having vaginal delivery showed that CCT was associated with reductions in the risk of blood loss ≥ 500 mL, mean volume of blood loss, and duration of the third stage of labour, with no significant difference in the risk of blood loss ≥ 1000 mL, need for additional uterotonics, or transfusion requirements. However, the risk of uterine inversion and cord avulsion should be noted and addressed by sufficient practical training [88].

A meta-analysis of randomised trials that compared manual removal and CCT for delivering the placenta at caesarean section revealed that CCT was associated with reductions in the risk of endometritis, volume of blood loss, change in postoperative haematocrit levels, and duration of hospital stay [89].

In term infants, delayed umbilical cord clamping is associated with increased Hb levels at birth, improved iron store in the first months of life, and higher mean birthweight compared with early cord clamping [90–92]. There is no significant difference in maternal outcomes (e.g. risk of PPH, mean blood loss, Hb levels at 24–72 h post-delivery, need for additional uterotonics) between early and delayed cord clamping [90–92].

- Statement 22. The use of oxytocin or carbetocin to prevent PPH during the 3^rd^ stage of labour is recommended for all births:

- Carbetocin is recommended in women undergoing caesarean delivery, and for those undergoing vaginal delivery who are at increased risk for PPH.
- Oxytocin is recommended in women undergoing vaginal delivery who do not have risk factors for PPH.

A recent meta-analysis of randomised trials that compared carbetocin and oxytocin in PPH prevention following low-risk caesarean delivery demonstrated that carbetocin was associated with reductions in the need for additional uterotonics and transfusions and a smaller drop in postoperative Hb levels, with no significant difference in the incidence of PPH or adverse effects [93]. Carbetocin offers a faster onset of action, more prolonged uterine contractions, more convenient administration (due to thermostability and a single-dose regimen), and acceptable cost-effectiveness, particularly in cases of high-risk delivery, compared with oxytocin [94, 95]. The panel noted that all private and public hospitals in Hong Kong employ prophylactic carbetocin in women undergoing caesarean delivery.

In the randomised CHAMPION trial of women giving vaginal birth, carbetocin was noninferior to oxytocin in preventing blood loss ≥ 500 mL or the need for additional uterotonics [96]. The panel considered that, in women undergoing vaginal delivery, carbetocin should be used to provide more rapid and long-acting effects in those at high risk for PPH, whereas oxytocin can be used in those with no known risk factors for PPH.

- Statement 23. The use of prophylactic TXA is recommended for high-risk patients with PPH.

In the randomised placebo-controlled TRAAP trial of women with vaginal delivery who received prophylactic oxytocin, the use of TXA did not significantly reduce the risk of PPH (≥ 500 mL measured with a collector bag; RR, 0.83; 95% CI, 0.68–1.01; *P* = 0.07), but significantly reduced the risk of provider-assessed clinically significant PPH (RR, 0.74; 95% CI, 0.61–0.91; *P* = 0.004; adjusted *P* = 0.04) and the need for additional uterotonics (RR, 0.75; 95% CI, 0.61–0.92; *P* = 0.006; adjusted *P* = 0.04) [97]. The authors suggested that TXA might be more likely to reduce the incidence of PPH among women with vaginal delivery that involved interventions (episiotomy or operative vaginal delivery), although the study was not powered to perform pertinent subgroup analyses [97].

The randomised placebo-controlled TRAAP2 trial included women who underwent caesarean delivery and received prophylactic uterotonics [98]. Compared with placebo, the use of TXA significantly reduced the risk of blood loss ≥ 1000 mL or receipt of a red-cell transfusion within 2 days after delivery (adjusted RR, 0.84; 95% CI, 0.75–0.94; *P* = 0.003), but did not significantly affect secondary outcomes related to haemorrhage [98]. A meta-analysis of randomised trials on the use of TXA in caesarean delivery concluded that there is a paucity of evidence to support the routine use of prophylactic TXA, especially among women at low risk of PPH [99].

Based on the current evidence, the panel highlighted the importance of patient selection when deciding to use prophylactic TXA for preventing PPH in vaginal or caesarean delivery.

- Statement 24. Second-line measures, including uterine compression sutures, balloon tamponade, and uterine artery embolisation, should be implemented early and with a lower threshold to help preventing PPH.

A retrospective cohort study in Hong Kong showed that, among patients with massive PPH (blood loss ≥ 1500 mL within 24 h post-delivery) that was uncontrolled using uterine massage and uterotonics, second-line therapies, including uterine compression sutures, uterine artery embolisation, and balloon tamponade, resulted in a low rate (21.4%) of rescue hysterectomy [100]. Additionally, an upward trend in the use of these second-line therapies was associated with a downward trend in the incidence of rescue hysterectomy and estimated blood loss [100].

Statement 25. Recommended treatments for PPH include the following:

- Uterine massage
- IV oxytocin alone as the first-line uterotonic treatment
- Second-line uterotonics (e.g. syntometrine, carboprost, misoprostol) if bleeding does not respond to oxytocin
- IV TXA administered as soon as possible (within 3 h) after bleeding onset, in addition to standard care
- Uterine balloon tamponade as a non-surgical treatment approach for PPH due to uterine atony if uterotonics are ineffective or unavailable
- Surgical intervention (e.g. compression suture, uterine and internal iliac artery ligation, hysterectomy) if PPH does not respond to uterotonics or other conservative treatments
- Single-unit transfusions may be considered
- Iron repletion may be considered

Uterine atony is the most common cause of PPH [101]. When blood loss is unresponsive to uterine massage, IV oxytocin should be the first-line treatment [101]. Notably, 3–25% of patients with PPH do not respond well to oxytocin and thus require a second-line uterotonic [102]. TXA is also an important therapy for PPH, as demonstrated in the randomised placebo-controlled WOMAN trial. In this study, it significantly reduced the risk of mortality due to bleeding (RR, 0.81; 95% CI, 0.65–1.00; *P* = 0.045), particularly when administered within 3 h post-delivery (RR, 0.69; 95% CI, 0.52–0.91; *P* = 0.008), in women with PPH following a vaginal or caesarean delivery [103]. Pooled data revealed the high success rate (85.9%) of uterine balloon tamponade for treating severe PPH, particularly in cases involving uterine atony (87.1%) and placenta praevia (86.8%) [104]. In high-income countries, the integration of uterine balloon tamponade into the management pathway for PPH has been shown to reduce the need for hysterectomy and PPH-related invasive procedures [104]. Treatment options for PPH-related anaemia include oral or IV iron therapy (especially when the oral formulation is intolerable, not well absorbed, or not well adhered to) and blood transfusion (when indicated, a single-unit approach is preferred to reduce the risk of transfusion-related adverse effects) [105, 106].

- Statement 26. Placenta accreta spectrum (PAS), often related to prior caesarean sections, is a major risk factor for massive PPH.
- Statement 27. In patients with PAS following caesarean sections, conservative management involving retention of the placenta *in situ* may be considered to minimise total blood loss, although the possibility of requiring hysterectomy cannot be ruled out.

PAS disorder comprises a range of conditions characterised by varying degrees of placental tissue adherence, ranging from abnormal attachment to severe invasion of the uterine wall [107]. The global incidence of PAS disorder is rising with the upward trend in the employment of caesarean sections, with both PAS disorder and caesarean sections being risk factors for massive PPH [107]. Based on experiences in public hospitals in Hong Kong, PAS disorder can be successfully treated with planned conservative management in an interdisciplinary approach involving interventional radiology [108, 109].

#### Patients on antithrombotic therapy

Statement 28. In patients on antithrombotic therapy (e.g. warfarin, DOACs, antiplatelets) who plan to undergo elective surgery, the decision to stop antithrombotic therapy should consider the following factors:

- Thrombotic risk associated with anticoagulation interruption in the perioperative period
- Surgery/procedure-related bleeding risk

Statement 29. Antithrombotic therapy can be continued in surgeries/procedures with minimal bleeding risk, whereas heparin bridging may be needed in patients taking warfarin and at high risk for thromboembolism during surgeries/procedures with bleeding risk (e.g. recent venous thromboembolism and mechanical heart valves).

Statement 30. Considering the wide variation in surgical procedures and patient comorbidities, thrombotic and bleeding risk assessment should be individualised and managed through a multidisciplinary approach.

In surgical populations, whether to continue or interrupt antithrombotic therapy depends on stratifications of thrombotic and bleeding risks. Notably, such stratifications are usually empiric and consider individual patient characteristics and surgical types [110, 111]. According to the American College of Chest Physicians Clinical Practice Guideline on the Perioperative Management of Antithrombotic Therapy, warfarin can be continued in surgeries with a minimal bleeding risk, whereas in surgeries with a low-to-moderate or high bleeding risk, warfarin should be interrupted preoperatively, and heparin bridging should be considered in patients at high thrombotic risk [111]. Discontinuation of DOACs is generally suggested before surgeries with a low-to-moderate or high bleeding risk [111]. With respect to antiplatelets, whether to continue or discontinue aspirin therapy depends on surgery or procedure bleeding risk assessment, while P2Y_12_ inhibitors should be interrupted before most surgeries [111].

## Part 3. Rationalising the use of blood and blood components

### Transfusion triggers

- Statement 1. In most surgical populations, there is no significant difference in clinical outcomes between restrictive and liberal transfusion strategies; however, restrictive strategies (i.e. lower Hb threshold and fewer transfusions of RBCs) aim to minimise unnecessary blood use and optimise patient safety by reducing transfusion risk.
- Statement 2. A restrictive transfusion threshold of Hb 7–8 g/dL is recommended for most surgical populations, including those undergoing non-cardiac surgery and those with critical conditions (e.g. patients admitted to the intensive care unit (ICU)).
- Statement 3. A transfusion threshold of Hb 8 g/dL is recommended for patients with stable cardiovascular disease. Consider a more liberal threshold (Hb 9–10 g/dL) for acute coronary syndrome (ACS) or anaemic heart failure.
- Statement 4. Transfusion thresholds should be individualised in patients receiving ECMO (VV-ECMO target Hb 7 g/dL (mainly for respiratory failure) VA-ECMO target Hb 8–9 g/dL (mainly for circulatory failure)), accounting for individual clinical status and context.

A transfusion trigger is the clinical threshold, primarily a low Hb level, that indicates the need for a blood transfusion to ensure sufficient oxygen delivery to organs and tissues. Despite the lack of a universally agreed definition, restrictive transfusion strategies typically allow for a higher tolerance of anaemia, initiating transfusions only when Hb levels drop to ~7 g/dL; in contrast, liberal transfusion strategies tend to commence transfusions at a higher threshold, e.g. Hb ~10 g/dL. When clinical outcomes are similar, a restrictive approach is preferred over a liberal approach to minimise unnecessary blood transfusions and associated adverse effects.

A meta-analysis included 48 randomised trials with 21,433 patients across varying clinical conditions (e.g. orthopaedic or cardiovascular surgery; critical care; acute blood loss (including gastrointestinal bleeding); ACS; cancers) [112]. Compared with liberal transfusion strategies (Hb threshold, 9.0–10.0 g/dL), restrictive transfusion strategies (Hb threshold, 7.0–8.0 g/dL) reduced the need for transfusions by 41% (RR, 0.59; 95% CI, 0.53–0.66; high-quality evidence), with no substantial impact on the risk of 30-day mortality (RR, 0.99; 95% CI, 0.86–1.15; moderate-quality evidence) or other outcomes assessed, including cardiac events (low-quality evidence), myocardial infarction, stroke, and thromboembolism (all high-quality evidence) [112]. Therefore, a restrictive transfusion threshold of Hb 7–8 g/dL should be considered for most surgical populations who are haemodynamically stable [106, 113, 114].

Although restrictive transfusion strategies appear generally favourable, their safety in populations with acute brain injury [115] or cardiovascular problems, or undergoing cardiovascular surgeries remains less certain [2, 106, 112, 116, 117].

A systematic review and frequentist-Bayesian meta-analysis included four randomised trials that compared liberal (Hb ≥ 9 g/dL) versus restrictive (Hb ≥ 7 g/dL) transfusion in adults with acute brain injury (i.e. traumatic brain injury, subarachnoid haemorrhage, and intracranial haemorrhage) and a Glasgow Coma Scale score ≤ 13 [115]. Pooled data from trials with a low risk of bias (with no heterogeneity, *I*^2^ = 0%) showed that the liberal strategy was associated with improved neurological outcomes (RR, 0.74; 95% CI, 0.63–0.87) [115].

Patients with coronary artery disease often have reduced physiological reserve that may necessitate a higher transfusion threshold. The randomised MINT trial demonstrated that, among patients with acute myocardial infarction and anaemia, restrictive transfusion (Hb 7–8 g/dL) appeared to be associated with a worse composite outcome of recurrent myocardial infarction or death at 30 days (RR, 1.15; 95% CI, 0.99–1.34; *P* = 0.07) compared with liberal transfusion (Hb 10 g/dL) [116]. A post hoc analysis of MINT assessed restrictive and liberal transfusion strategies among patients with and without baseline heart failure [117]. The rate ratio of death or heart failure at 30 days between restrictive and liberal transfusion was numerically higher in those with baseline heart failure than in those without (RR, 1.20 [95% CI, 0.99–1.45] vs. 0.94 [95% CI, 0.70–1.26]; *P*_interaction_ = 0.18) [117].

Patients with ECMO require individualised transfusion thresholds. A retrospective study in Hong Kong showed that, in a cohort of patients with ECMO (VV, 30%; VA, 50%), using transfusion thresholds of Hb ≤ 8.5 g/dL and > 8.5 g/dL yielded no significant differences in ICU mortality (adjusted odds ratio (OR), 0.86; 95% CI, 0.56–1.30; *P* = 0.47), hospital mortality (adjusted OR, 0.79; 95% CI, 0.52–1.21; *P* = 0.28), or 90-day mortality (adjusted OR, 0.84; 95% CI, 0.55–1.28; *P* = 0.42) [118]. In the subgroup of patients with ECMO-VV, Hb ≤ 8.5 g/dL (vs. > 8.5 g/dL) was associated with a reduced risk of ICU mortality (adjusted OR, 0.36; 95% CI, 0.17–0.73; *P* = 0.005) [118]. The international prospective observational PROTECMO study demonstrated that, among patients with ECMO-VV (mean pretransfusion Hb, 8.1 g/dL), Hb < 7 g/dL was associated with a higher risk of death in the ICU (HR, 2.99; 95% CI, 1.95–4.60) compared with higher Hb levels, while packed RBC transfusion was associated with a lower risk of mortality (HR, 0.15; 95% CI, 0.03–0.74) only when administered with Hb < 7 g/dL (other Hb thresholds: 7.0–7.9, 8.0–9.9, and > 10 g/dL) [119].

- Statement 5. When indicated, single-unit transfusions with reassessment should replace multiunit orders.

In patients who are haemodynamically stable and not actively bleeding, single-unit transfusions, followed by clinical reassessments before the administration of additional units, should be considered, because this strategy is often adequate to restore oxygen delivery and treat anaemia, while offering the potential benefits of reducing unnecessary transfusions, risks of transfusion-related complications, and healthcare costs [113, 120, 121].

- Statement 6. Transfusion strategies should be adjusted based on patient-specific factors, including laboratory data, clinical context, symptoms, and signs (Table 2), in addition to Hb levels.

Notably, restrictive and single-unit transfusions may not be suitable in patients who are haemodynamically unstable, hypovolaemic, actively bleeding, or severely anaemic. Rather than a routine practice, these strategies should be implemented in specific patient groups after consideration of individual patient factors and conditions.

- Statement 7. Routine use of physiologic transfusion triggers (e.g. systemic oxygen delivery, ST segment changes on electrocardiogram, mixed venous oxygen saturation, lactate levels, near-infrared spectroscopy) is not recommended because of a lack of high-level evidence.

The Hb level remains the standard transfusion trigger that facilitates continuous monitoring of anaemic status and thus timely transfusions. Alternatively, there are a range of alternative blood transfusion triggers that are mainly based on the physiological parameters of oxygen delivery/consumption ratio, attempting to address the insufficiency of the isolated Hb level in reflecting oxygen delivery [122]. Although physiological triggers are theoretically optimal to determine the need for transfusions, their use in routine clinical practice should be further verified in large-scale randomised trials [123].

### Implementation of transfusion protocols

Statement 8. Suggested key components of a hospital-wide transfusion protocol include the following:

- Clear Hb thresholds tailored to patient subgroups
- Mandatory Hb check before transfusions when clinical context allows, and with application of POC coagulation testing as necessary
- Assessment of coagulation, biochemical and metabolic parameters
- Mandatory assessment of patient conditions before transfusion
- Single-unit transfusion orders as far as possible
- Checklists to ensure reassessments after single-unit transfusions
- Monitoring for adverse effects (e.g. ischaemic complications, haemodynamic instability, organ dysfunction)

The panel highlighted the importance of developing a hospital-wide transfusion protocol with the above items. The principle is to facilitate efficient and safe transfusions via mandatory assessments of Hb levels and clinical conditions (e.g. active bleeding, ischaemic symptoms), implementation of the single-unit policy (investigation of abnormalities, e.g. occult bleeding and hypoxia, may be considered when additional units are required), and monitoring for adverse effects (e.g. troponin rise, changes in electrocardiogram, tachycardia, hypotension, acute kidney injury, altered mental status).

Statement 9. Suggested measures to facilitate the implementation of transfusion strategies include the following:

- Staff training on transfusion indications, restrictive thresholds, and the single-unit policy
- Electronic reminders to confirm the need for transfusion in patients with Hb 7–8 g/dL without symptoms
- Transfusion audits to monitor compliance, transfusion reactions, and clinical outcomes
- Feedback and refreshment training for clinicians based on audit data
- Refinement of protocols based on audit data

Staff training and decision-support tools, e.g. electronic alerts for Hb thresholds and checklists to ensure reassessment following a single-unit transfusion, are crucial to facilitate the implementation of a transfusion protocol. Audits should be conducted to monitor protocol compliance and a range of metrics, including the percentage of single-unit transfusions, incidences of transfusion-related reactions, and clinical outcomes (e.g. mortality rates, length of hospital stay, complications). Protocols can be refined based on audit results.

### Advantages and challenges of PBM programmes

- Statement 10. PBM programmes consistently deliver healthcare savings via improvements in patient outcomes, including shortening hospital stays, reducing postoperative complications, and minimising unnecessary blood transfusions.
- Statement 11. PBM programmes enhance resource sustainability and ethical use of blood products.

PBM programmes that include preoperative screening for anaemia and suboptimal iron stores, perioperative bleeding reduction measures, and restrictive transfusion strategies are generally considered to be cost-effective (reducing unnecessary transfusions and related complications) and to improve patient outcomes [124–127]. A retrospective study showed that, over 6 years, a jurisdiction-wide PBM programme initiated across four major hospitals in Western Australia significantly reduced the hospital mortality rate by 28%, average length of hospital stays by 15%, incidence of hospital-acquired infections by 21%, incidence of heart attack or stroke by 31%, and units of transfusions per admission by 41%, translating to substantial cost savings [127].

In addition to being cost-effective, PBM programmes support the ethical use of blood products by minimising the need for transfusions and improving patient outcomes, considering their limited availability, reliance on voluntary donation, and inherent worth [125].

- Statement 12. Disease- and specialty-specific protocols are needed to consistently implement PBM programmes.
- Statement 13. Key challenges and barriers towards the implementation of PBM include hospital culture, inadequate staff awareness, poor interdisciplinary communication or collaboration, absence of electronic monitoring systems, and resource limitations (staff, time, and finances).

Consistent implementation of a hospital-based PBM programme requires dedicated protocols that address perioperative bleeding reduction interventions, Hb thresholds for transfusions, and the application of single-unit transfusions for different surgical populations. Notably, more local research is warranted to justify enhancements in investments and resources to help resolve the challenges and barriers towards implementation of PBM programmes [1].

## Conclusion

The HKSCBM updated its consensus statements to facilitate the implementation of PBM across three pillars: optimisation of RBC mass and anaemia management, minimisation of perioperative blood loss, and rational use of blood and blood components. The consensus panel’s multidisciplinary composition addressed measures to enhance PBM practices for various surgical populations, including obstetric, gynaecologic, orthopaedic, renal, gastrointestinal, cardiovascular, oncological, emergency, traumatic, and elderly patients. These statements are intended to serve as a practical framework for clinicians and healthcare providers involved in PBM.

While recognising the importance of patient empowerment in PBM, the panel did not include patient, caregiver, or public representatives in the present consensus process. This consensus primarily focused on establishing clinician-directed recommendations and promoting implementation among healthcare professionals. Further panel initiatives will aim to enhance public awareness through patient education, engagement with patient advocacy groups, and incorporation of these perspectives into subsequent consensus development. The HKSCBM will continue to advance research and cooperate with relevant stakeholders, including hospital administrators, medical and surgical colleagues, and patients, to promote optimal PBM practices across divisions, departments, and hospitals. Notably, PBM practices for other highly specialised areas, such as cardiothoracic surgery and paediatrics, will be addressed in future updates by expanding the consensus panel to include related experts.

## Supplementary Information

Additional file 1. ACCORD checklist. Description: Completion of the checklist demonstrated that this consensus-based research was reported as per the ACCORD guidelines. 

Additional file 2. Full voting records for all statements. Description: The file tabulates the detailed voting results for each accepted and rejected statement.

## Data Availability

All data generated or analysed during this study are included in this published article and its supplementary information file.

## Abbreviations

ACS: Acute coronary syndrome
CBC: Complete blood count
CCT: Controlled cord traction
CI: Confidence interval
CKD: Chronic kidney disease
DOAC: Direct oral anticoagulant
ECMO: Extracorporeal membrane oxygenation
ESA: Erythropoiesis-stimulating agent
GnRH: Gonadotropin-releasing hormone
Hb: Haemoglobin
HKSCBM: Hong Kong Society of Clinical Blood Management
HR: Hazard ratio
ICU: Intensive care unit
IDA: Iron deficiency anaemia
INR: International normalised ratio
IV: Intravenous
IVC: Inferior vena cava
KDIGO: Kidney Disease: Improving Global Outcomes
OR: Odds ratio
PAS: Placenta accreta spectrum
PBM: Patient blood management
PCC: Prothrombin complex concentrate
POC: Point-of-care
PPH: Postpartum haemorrhage
RBC: Red blood cell
ROTEM: Rotational thromboelastometry
RR: Risk ratio
TEG: Thromboelastography
TSAT: Transferrin saturation
TXA: Tranexamic acid
VA: Venoarterial
VHA: Viscoelastic haemostatic assay
VV: Venovenous
WHO: World Health Organisation
