# Supplementary material for: Recommendations for implementing patient blood management—an updated modified Delphi consensus from a multidisciplinary expert panel in Hong Kong
Source: J Anesth Analg Crit Care. 2026 May 26;6:78. doi: 10.1186/s44158-026-00417-4 (PMC13202949; doi:10.1186/s44158-026-00417-4)
Supplement: Supplementary file 2 — Additional file 2. Full voting records for all statements. Description: The file tabulates the detailed voting results for each accepted and rejected statement. [file 44158_2026_417_MOESM2_ESM.docx]

**Additional file 2. Full voting records for all statements**

| **Part 1 – Optimising patients’ red blood cell mass and improving anaemia management** | | | | | | | | | | |  |
| --- | --- | --- | --- | --- | --- | --- | --- | --- | --- | --- | --- |
| **A. Groups at high risk for iron deficiency and anaemia** | | **Response options* (%)** | | | | | | **Statement was accepted only if  (A + B)% ≥ 80%** | | |  |
| **#** | **Drafted statements** | | **A** | **B** | **C** | **D** | **E** | | **A + B (%)** | **Accepted/Rejected** | |
| **1** | Groups at high risk for iron deficiency and anaemia include: | |  | | | | | | | | |
| **1a** | Women at reproductive age. | | 100 | / | / | / | / | | 100 | ✓ | |
| **1b** | Antenatal or postnatal women. | | 100 | / | / | / | / | | 100 | ✓ | |
| **1c** | Patients with cancer. | | 100 | / | / | / | / | | 100 | ✓ | |
| **1d** | Patients with heart disease. | | 92 | 8 | / | / | / | | 100 | ✓ | |
| **1e** | Patients with bowel disorders (e.g. peptic ulcer, inflammatory bowel disease). | | 100 | / | / | / | / | | 100 | ✓ | |
| **1f** | Patients with renal disease. | | 100 | / | / | / | / | | 100 | ✓ | |
| **1g** | Elderly populations. | | 92 | 8 | / | / | / | | 100 | ✓ | |
| **1h** | Patients on extracorporeal membrane oxygenation (ECMO). | | 92 | 8 | / | / | / | | 100 | ✓ | |
| **2** | Other conditions that are prone to adverse effects of iron deficiency anaemia (IDA): | |  | | | | | | | | |
| **2a** | Acute brain injury. | | 46 | 54 | / | / | / | | 100 | ✓ | |
| **2b** | Septic shock. | | 54 | 46 | / | / | / | | 100 | ✓ | |
| **B. Preoperative screening for IDA** | |  | | | | | | | | |  |
| **3** | For surgical populations, the optimal preoperative haemoglobin (Hb) level should be: | |  | | | | | | | | |
| **3a** | ≥ 13.0 g/dL in both males and females. | | 8 | 38 | 46 | 8 | / | | 46 | ✘ | |
| **3b** | ≥ 13.0 g/dL in males and ≥ 12.0 g/dL in females. | | 62 | 31 | 8 | / | / | | 92 | ✓ | |
| **4** | All surgical populations should be routinely screened for anaemia, focusing on absolute or functional iron deficiency. | | 85 | 15 | / | / | / | | 100 | ✓ | |
| **5** | Preoperative screening for IDA facilitates diagnosis and earlier administration of oral or intravenous iron therapy, thereby reducing hospitalisations for blood transfusions and contributing to higher rates of same-day surgery. | | 85 | 15 | / | / | / | | 100 | ✓ | |
| **6** | It may be worthwhile to adopt preoperative protocol-driven evaluation for IDA. | | 100 | / | / | / | / | | 100 | ✓ | |
| **7** | Hospital- or department-specific algorithms should be established to govern preoperative screening and management of IDA, including the roles of nurses, anaesthesiologists, physicians, haematopathologists, and surgeons. | | 100 | / | / | / | / | | 100 | ✓ | |
| **8** | A nurse-led clinic can be implemented to facilitate preoperative screening for IDA, with the roles including: | |  | | | | | | | | |
| **8a** | Interpretation of protocol-driven anaemia evaluation. | | 92 | 8 | / | / | / | | 100 | ✓ | |
| **8b** | Patient education on iron deficiency treatments, including effectiveness and tolerability of oral and intravenous iron therapies. | | 100 | / | / | / | / | | 100 | ✓ | |
| **8c** | Administration of intravenous iron therapy when necessary, using dosing tools. | | 69 | 31 | / | / | / | | 100 | ✓ | |
| **8d** | Alert attending surgeons and anaesthesiologists to unexplained or severe anaemia cases, and consider referral to physicians for further work-up. | | 100 | / | / | / | / | | 100 | ✓ | |
| **9** | Clinical management systems should be enhanced to track patient outcomes and automatically notify attending surgeons and anaesthesiologists if rechecked Hb remains below the target within 7–10 days before surgery. | | 77 | 23 | / | / | / | | 100 | ✓ | |
| **10** | Further local research should be conducted to assess the prevalence of anaemia and their causes among surgical populations in Hong Kong. | | 100 | / | / | / | / | | 100 | ✓ | |
| **C. Preoperative measures to optimise Hb levels in the general population** | |  | | | | | | | | |  |
| ***C1. Patients undergoing elective surgery*** | |  | | | | | | | | |  |
| **11** | In patients with suboptimal preoperative Hb levels, delay of elective surgery should be considered, and an iron supplement should be administered to correct the deficiency while investigating the underlying causes. | | 69 | 23 | 8 | / | / | | 92 | ✓ | |
| **12** | Transfusions should be avoided in patients undergoing elective surgery when their IDA is clinically stable and correctable. | | 77 | 23 | / | / | / | | 100 | ✓ | |
| ***C2. Patients undergoing urgent surgery*** | |  | | | | | | | | |  |
| **13** | In patients who require more urgent surgery (e.g. within 2–4 weeks), preoperative IDA should be treated with intravenous iron therapy. | | 77 | 23 | / | / | / | | 100 | ✓ | |
| **14** | To treat preoperative anaemia in patients who are clinically unstable or require urgent surgery, transfusions (preferably with a restrictive Hb threshold and a single-unit approach [refer to Part 3]) may be considered. | | 85 | 8 | 8 | / | / | | 92 | ✓ | |
| **D. Preoperative measures to optimise Hb levels in special populations** | |  | | | | | | | | |  |
| ***D1. Gynaecological patients*** | |  | | | | | | | | |  |
| **15** | In patients with heavy menstrual bleeding and severe anaemia (i.e. Hb < 7 g/dL) due to fibroids: | |  | | | | | | | | |
| **15a** | Preoperative use of gonadotropin-releasing hormone (GnRH) agonists for 3–4 months can be considered to reduce intraoperative blood loss. | | 54 | 46 | / | / | / | | 100 | ✓ | |
| **15b** | Intravenous iron therapy ± a single-unit transfusion can be considered to optimise preoperative Hb levels, provided the patient is haemodynamically stable. | | 85 | 15 | / | / | / | | 100 | ✓ | |
| **15c** | Early surgery should be considered in those who have already planned for the procedure. | | 85 | 15 | / | / | / | | 100 | ✓ | |
| ***D2. Obstetric patients*** | |  | | | | | | | | |  |
| **16** | In antenatal patients, screening of serum ferritin levels can be considered for early detection of IDA. | | 100 | / | / | / | / | | 100 | ✓ | |
| **17** | The following treatment measures can be considered in antenatal patients: | |  | | | | | | | | |
| **17a** | Iron-rich diet. | | 85 | 15 | / | / | / | | 100 | ✓ | |
| **17b** | Oral iron supplementation. | | 92 | 8 | / | / | / | | 100 | ✓ | |
| **17c** | Intravenous iron therapy in the 2^nd^ or 3^rd^ trimesters (especially when oral iron therapy is contraindicated, intolerable, or ineffective). | | 100 | / | / | / | / | | 100 | ✓ | |
| **18** | Regarding the administration of intravenous iron therapy, the need for monitoring for any potential adverse reactions under an institutional protocol should be discussed with parturients in advance. | | 85 | 15 | / | / | / | | 100 | ✓ | |
| ***D3. Renal patients*** | |  | | | | | | | | |  |
| **19** | Higher thresholds (i.e. serum ferritin ≤ 800 ng/mL, transferrin saturation < 30%, and ferritin < 500 ng/mL) could increase the sensitivity of identifying IDA in patients with chronic kidney disease. | | 77 | 23 | / | / | / | | 100 | ✓ | |
| **20** | To treat anaemia in patients with chronic kidney disease: | |  | | | | | | | | |
| **20a** | Intravenous iron therapy is preferred over oral iron therapy, especially when response to oral iron therapy is suboptimal. | | 38 | 62 | / | / | / | | 100 | ✓ | |
| **20b** | Erythropoiesis-stimulating agents can be considered in selected cases. | | 100 | / | / | / | / | | 100 | ✓ | |
| **20c** | A conservative Hb target should be maintained. | | 85 | 15 | / | / | / | | 100 | ✓ | |
| **20d** | Coordination with nephrologists should be considered when necessary. | | 100 | / | / | / | / | | 100 | ✓ | |
| ***D4. Patients with bowel disorders*** | |  | | | | | | | | |  |
| **21** | Screening for haematinics deficiency, including iron profile, vitamin B12 and folate, is recommended. | | 85 | 8 | 8 | / | / | | 92 | ✓ | |
| **22** | Intravenous iron therapy, instead of oral iron therapy, is recommended in patients with iron deficiency due to malabsorption by the gastrointestinal tract. | | 85 | 15 | / | / | / | | 100 | ✓ | |
| ***D5. Elderly patients*** | |  | | | | | | | | |  |
| **23** | Routine screening for medications that pose a high risk for anaemia is recommended. | | 85 | 8 | 8 | / | / | | 92 | ✓ | |
| **24** | Intravenous iron therapy may be preferred over oral iron therapy in patients with polypharmacy. | | 69 | 31 | / | / | / | | 100 | ✓ | |
| **E. Patient empowerment** | |  | | | | | | | | |  |
| **25** | Public education in the primary and community healthcare settings should be enhanced to raise awareness of IDA, especially among women at reproductive age. | | 100 | / | / | / | / | | 100 | ✓ | |
| **26** | Patients should always be counselled regarding risks associated with anaemia and blood transfusions. | | 100 | / | / | / | / | | 100 | ✓ | |
| **27** | Patient preferences, acceptance, or rejection regarding blood components and/or blood conservation modalities should be discussed preoperatively. | | 100 | / | / | / | / | | 100 | ✓ | |
| **28** | Related consent forms and advanced directives should be obtained and documented preoperatively to ensure that acceptable options for optimal care are provided. | | 92 | 8 | / | / | / | | 100 | ✓ | |

*Response options: A, accept completely; B, accept with some reservation; C, accept with major reservation; D, reject with reservation; and E, reject completely.

| **Part 2. Minimising perioperative blood loss** | | | | | | | | | | |
| --- | --- | --- | --- | --- | --- | --- | --- | --- | --- | --- |
| **A. Surgeries and conditions associated with substantial blood loss** | | **Response options* (%)** | | | | | | **Statement was accepted only if  (A + B)% ≥ 80%** | | |
| **#** | **Drafted statements** | **A** | **B** | **C** | **D** | **E** | **A + B (%)** | | **Accepted/Rejected** |  |
| **1** | Perioperative bleeding is common in diverse surgical fields, including: |  | | | | | | | | |
| **1a** | Trauma surgery. | 100 | / | / | / | / | 100 | | ✓ |  |
| **1b** | Orthopaedic surgery. | 100 | / | / | / | / | 100 | | ✓ |  |
| **1c** | Neurosurgery. | 62 | 38 | / | / | / | 100 | | ✓ |  |
| **1d** | Visceral and transplant surgery. | 92 | 8 | / | / | / | 100 | | ✓ |  |
| **1e** | Cardiac and vascular surgery. | 100 | / | / | / | / | 100 | | ✓ |  |
| **1f** | Obstetric and gynaecological surgery. | 100 | / | / | / | / | 100 | | ✓ |  |
| **2** | In the preoperative phase, interventions should be initiated to identify patients who may be at higher risk for bleeding, including those with: |  | | | | | | | | |
| **2a** | Underlying coagulation abnormalities (inherited or acquired). | 100 | / | / | / | / | 100 | | ✓ |  |
| **2b** | Antithrombotic medications. | 100 | / | / | / | / | 100 | | ✓ |  |
| **B. Measures to minimise intraoperative blood loss in the general population** | |  | | | | | | | | |
| ***B1. Surgical techniques*** | |  | | | | | | | | |
| **3** | Judicious use of minimally invasive surgery, electrocautery, tourniquets, topical haemostatic agents (including mechanical and active biologic agents), and intraoperative blood salvage should be considered to reduce blood loss. | 85 | 15 | / | / | / | 100 | | ✓ |  |
| ***B2. Patient positioning*** | |  | | | | | | | | |
| **4** | Correct patient positioning is a simple and effective intervention to minimise intraoperative blood loss. | 92 | 8 | / | / | / | 100 | | ✓ |  |
| **5** | The general principles of patient positioning include elevation of surgical sites and slow transition of positions. | 92 | 8 | / | / | / | 100 | | ✓ |  |
| ***B3. Normothermia*** | |  | | | | | | | | |
| **6** | Maintaining perioperative normothermia is crucial to reduce blood loss and the need for blood transfusions. | 100 | / | / | / | / | 100 | | ✓ |  |
| ***B4. Point-of-care (POC) coagulation testing*** | |  | | | | | | | | |
| **7** | POC testing of blood coagulation using a viscoelastic haemostatic assay (e.g. rotational thromboelastometry or thromboelastography) or ultrasound-induced resonance helps reducing the requirement for blood product transfusions by guiding the haemostatic therapy. | 92 | 8 | / | / | / | 100 | | ✓ |  |
| **8** | POC testing of blood coagulation is recommended in cases of suspected coagulopathy or surgical settings where massive haemorrhage is anticipated (e.g. trauma, cardiac, liver, obstetric and gynaecological surgery) | 100 | / | / | / | / | 100 | | ✓ |  |
| ***B5. Haemostatic agents*** | |  | | | | | | | | |
| **9** | Tranexamic acid can be administered perioperatively to reduce the risk of major bleeding in non-cardiac surgeries. | 100 | / | / | / | / | 100 | | ✓ |  |
| **10** | Fibrinogen concentrate helps minimising intraoperative blood loss in certain surgical populations, including cardiac surgery, massive obstetric haemorrhage, or polytrauma with severe bleeding. | 100 | / | / | / | / | 100 | | ✓ |  |
| **11** | In patients with expected massive haemorrhage awaiting viscoelastic or laboratory tests, administering 2-g fibrinogen concentrate based on clinical criteria at admission—such as low systolic blood pressure, metabolic acidosis, or low Hb levels—helps to provide initial coagulation support and correct hypofibrinogenemia. | 77 | 23 | / | / | / | 100 | | ✓ |  |
| ***B6. Reversal agents for anticoagulation*** | |  | | | | | | | | |
| **12** | Prothrombin complex concentrate (PCC) and vitamin K1 are indicated for the urgent reversal of anticoagulation in patients with major acute bleeding, such as intracerebral haemorrhage, or those requiring emergency surgery who are taking warfarin. | 85 | 15 | / | / | / | 100 | | ✓ |  |
| **13** | Regarding the use of specific reversal agents: |  | | | | | | | | |
| **13a** | Idarucizumab should be considered in patients taking dabigatran if urgent reversal of anticoagulation is indicated; for example, in major acute bleeding or before emergency surgery. | 85 | 15 | / | / | / | 100 | | ✓ |  |
| **13b** | Andexanet alfa, the reversal agent for factor Xa inhibitors (e.g. rivaroxaban, apixaban, and edoxaban), is not readily available in Hong Kong. If rapid reversal of an oral factor Xa inhibitor is indicated, PCC could be considered. | 85 | 15 | / | / | / | 100 | | ✓ |  |
| **14** | PCC may serve as a treatment option for acute massive haemorrhage (not warfarin-induced) in patients undergoing surgery. | 38 | 54 | 8 | / | / | 92 | | ✓ |  |
| **15** | In acute massive haemorrhage, the advantages of PCC over plasma may include faster onset of action, off-the-shelf availability, no thawing requirement, and reduced risk of fluid overload. | 92 | 8 | / | / | / | 100 | | ✓ |  |
| **16** | The limitations of PCC in the management of acute massive haemorrhage include the inability to replenish all clotting factors and the thrombotic risk associated with an overdose. Its use is preferably guided by POC coagulation testing. | 85 | 15 | / | / | / | 100 | | ✓ |  |
| ***B7. Advanced haemodynamic monitoring*** | |  | | | | | | | | |
| **17** | In surgical populations at high risk for massive bleeding, advanced haemodynamic monitoring providing continuous data (e.g. cardiac output, fluid responsiveness) can be considered to facilitate targeted and timely interventions, thereby minimising intraoperative blood loss and the need for blood transfusions, and improving patient outcomes. | 92 | 8 | / | / | / | 100 | | ✓ |  |
| **C. Measures to minimise intraoperative blood loss in special populations** | |  | | | | | | | | |
| ***C1. Orthopaedic patients*** | |  | | | | | | | | |
| **18** | During prone spine surgery, the abdomen should be well positioned to avoid compressing the inferior vena cava. | 100 | / | / | / | / | 100 | | ✓ |  |
| **19** | Closed suction drains are not recommended in hip and knee arthroplasty. | 69 | 31 | / | / | / | 100 | | ✓ |  |
| ***C2. Obstetric patients*** | |  | | | | | | | | |
| **20** | Prevention of postpartum haemorrhage (PPH; defined as a blood loss of ≥ 500 mL within 24 hours after vaginal or caesarean delivery) is important in obstetric populations. | 100 | / | / | / | / | 100 | | ✓ |  |
| **21** | To reduce the risk of PPH, the umbilical cord can be managed as follows: |  | | | | | | | | |
| **21a** | For vaginal delivery, controlled cord traction can be offered routinely during the 3^rd^ stage of labour, provided that the birth attendant has the necessary skills. | 100 | / | / | / | / | 100 | | ✓ |  |
| **21b** | For caesarean delivery, controlled cord traction is recommended for the removal of the placenta. | 100 | / | / | / | / | 100 | | ✓ |  |
| **21c** | Delayed (instead of early) cord clamping is recommended for all births unless the neonate is asphyxiated and needs to be moved immediately for resuscitation. | 100 | / | / | / | / | 100 | | ✓ |  |
| **22** | The use of oxytocin or carbetocin to prevent PPH during the 3^rd^ stage of labour is recommended for all births: |  | | | | | | | | |
| **22a** | Carbetocin is recommended in women undergoing caesarean delivery, and for those undergoing vaginal delivery who are at increased risk for PPH. | 100 | / | / | / | / | 100 | | ✓ |  |
| **22b** | Oxytocin is recommended in women undergoing vaginal delivery who do not have risk factors for PPH. | 85 | 15 | / | / | / | 100 | | ✓ |  |
| **23** | The use of prophylactic tranexamic acid is recommended for high-risk patients with PPH. | 100 | / | / | / | / | 100 | | ✓ |  |
| **24** | Second-line measures, including uterine compression sutures, balloon tamponade, and uterine artery embolisation, should be implemented early and with a lower threshold to help preventing PPH. | 100 | / | / | / | / | 100 | | ✓ |  |
| **25** | Recommended treatments for PPH include: |  | | | | | | | | |
| **25a** | Uterine massage. | 100 | / | / | / | / | 100 | | ✓ |  |
| **25b** | Intravenous oxytocin alone as the first-line uterotonic treatment. | 69 | 31 | / | / | / | 100 | | ✓ |  |
| **25c** | Second-line uterotonics (e.g. syntometrine, carboprost, misoprostol) if bleeding does not respond to oxytocin. | 92 | 8 | / | / | / | 100 | | ✓ |  |
| **25d** | Intravenous tranexamic acid administered as soon as possible (within 3 hours) after bleeding onset, in addition to standard care. | 100 | / | / | / | / | 100 | | ✓ |  |
| **25e** | Uterine balloon tamponade as a non-surgical treatment approach for PPH due to uterine atony if uterotonics are ineffective or unavailable. | 100 | / | / | / | / | 100 | | ✓ |  |
| **25f** | Surgical intervention (e.g. compression suture, uterine and internal iliac artery ligation, hysterectomy) if PPH does not respond to uterotonics or other conservative treatments. | 100 | / | / | / | / | 100 | | ✓ |  |
| **25g** | Single-unit transfusions may be considered. | 46 | 38 | 15 | / | / | 85 | | ✓ |  |
| **25h** | Iron repletion may be considered. | 77 | 8 | 15 | / | / | 85 | | ✓ |  |
| **26** | Placenta accreta spectrum, often related to prior caesarean sections, is a major risk factor for massive PPH. | 100 | / | / | / | / | 100 | | ✓ |  |
| **27** | In patients with placenta accreta spectrum following caesarean sections, conservative management involving retention of the placenta *in situ* may be considered to minimise total blood loss, although the possibility of requiring hysterectomy cannot be ruled out. | 92 | 8 | / | / | / | 100 | | ✓ |  |
| ***C3. Patients on antithrombotic therapy*** | |  | | | | | | | | |
| **28** | In patients on antithrombotic therapy (e.g. warfarin, direct oral anticoagulants, antiplatelets) who plan to undergo elective surgery, the decision to stop antithrombotic therapy should consider the following factors: |  | | | | | | | | |
| **28a** | Thrombotic risk associated with anticoagulation interruption in the perioperative period. | 100 | / | / | / | / | 100 | | ✓ |  |
| **28b** | Surgery/procedure-related bleeding risk. | 92 | 8 | / | / | / | 100 | | ✓ |  |
| **29** | Antithrombotic therapy can be continued in surgeries/procedures with minimal bleeding risk, whereas heparin bridging may be needed in patients taking warfarin and at high risk for thromboembolism during surgeries/procedures with bleeding risk (e.g. recent venous thromboembolism and mechanical heart valves). | 69 | 23 | 8 | / | / | 92 | | ✓ |  |
| **30** | Considering the wide variation in surgical procedures and patient comorbidities, thrombotic and bleeding risk assessment should be individualised and managed through a multidisciplinary approach. | 100 | / | / | / | / | 100 | | ✓ |  |

*Response options: A, accept completely; B, accept with some reservation; C, accept with major reservation; D, reject with reservation; and E, reject completely.

| **Part 3. Rationalising the use of blood and blood components** | | | | | | | | |
| --- | --- | --- | --- | --- | --- | --- | --- | --- |
| **A. Transfusion triggers** | | **Response options* (%)** | | | | | **Statement was accepted only if  (A + B)% ≥ 80%** | |
| **#** | **Drafted statements** | **A** | **B** | **C** | **D** | **E** | **A + B (%)** | **Accepted/Rejected** |
| **1** | In most surgical populations, there is no significant difference in clinical outcomes between restrictive and liberal transfusion strategies; however, restrictive strategies (i.e. lower Hb threshold and fewer transfusions of red blood cells) aim to minimise unnecessary blood use and optimise patient safety by reducing transfusion risk. | 77 | 23 | / | / | / | 100 | ✓ |
| **2** | A restrictive transfusion threshold of Hb 7–8 g/dL is recommended for most surgical populations, including those undergoing non-cardiac surgery and those with critical conditions (e.g. patients admitted to the intensive care unit). | 62 | 38 | / | / | / | 100 | ✓ |
| **3** | A transfusion threshold of Hb 8 g/dL is recommended for patients with stable cardiovascular disease. Consider a more liberal threshold (Hb 9–10 g/dL) for acute coronary syndrome or anaemic heart failure. | 92 | / | 8 | / | / | 92 | ✓ |
| **4** | Transfusion thresholds should be individualised in patients receiving ECMO (venovenous-ECMO target Hb 7 g/dL [mainly for respiratory failure]; venoarterial-ECMO target Hb 8–9 g/dL [mainly for circulatory failure]), accounting for individual clinical status and context. | 69 | 31 | / | / | / | 100 | ✓ |
| **5** | When indicated, single-unit transfusions with reassessment should replace multiunit orders. | 100 | / | / | / | / | 100 | ✓ |
| **6** | Transfusion strategies should be adjusted based on patient-specific factors, including laboratory data, clinical context, symptoms, and signs, in addition to Hb levels. | 92 | 8 | / | / | / | 100 | ✓ |
| **7** | Routine use of physiologic transfusion triggers (e.g. systemic oxygen delivery, ST segment changes on electrocardiogram, mixed venous oxygen saturation, lactate levels, near-infrared spectroscopy) is not recommended because of a lack of high-level evidence. | 54 | 46 | / | / | / | 100 | ✓ |
| **B. Implementation of transfusion protocols** | |  | | | | | | |
| **8** | Suggested key components of a hospital-wide transfusion protocol include: |  | | | | | | |
| **8a** | Clear Hb thresholds tailored to patient subgroups. | 85 | 15 | / | / | / | 100 | ✓ |
| **8b** | Mandatory Hb check before transfusions when clinical context allows, and with application of POC coagulation testing as necessary. | 62 | 38 | / | / | / | 100 | ✓ |
| **8c** | Assessment of coagulation, biochemical and metabolic parameters. | 69 | 23 | 8 | / | / | 92 | ✓ |
| **8d** | Mandatory assessment of patient conditions before transfusion. | 100 | / | / | / | / | 100 | ✓ |
| **8e** | Single-unit transfusion orders as far as possible. | 85 | 15 | / | / | / | 100 | ✓ |
| **8f** | Checklists to ensure reassessments after single-unit transfusions | 85 | 15 | / | / | / | 100 | ✓ |
| **8g** | Monitoring for adverse effects (e.g. ischaemic complications, haemodynamic instability, organ dysfunction) | 92 | 8 | / | / | / | 100 | ✓ |
| **9** | Suggested measures to facilitate the implementation of transfusion strategies include: |  | | | | | | |
| **9a** | Staff training on transfusion indications, restrictive thresholds, and the single-unit policy. | 92 | 8 | / | / | / | 100 | ✓ |
| **9b** | Electronic reminders to confirm the need for transfusion in patients with Hb 7–8 g/dL without symptoms. | 54 | 38 | 8 | / | / | 92 | ✓ |
| **9c** | Transfusion audits to monitor compliance, transfusion reactions, and clinical outcomes. | 92 | 8 | / | / | / | 100 | ✓ |
| **9d** | Feedback and refreshment training for clinicians based on audit data. | 100 | / | / | / | / | 100 | ✓ |
| **9e** | Refinement of protocols based on audit data. | 85 | 15 | / | / | / | 100 | ✓ |
| **C. Advantages and challenges of patient blood management (PBM) programmes** | |  | | | | | | |
| **10** | PBM programmes consistently deliver healthcare savings via improvements in patient outcomes, including shortening hospital stays, reducing postoperative complications, and minimising unnecessary blood transfusions. | 92 | 8 | / | / | / | 100 | ✓ |
| **11** | PBM programmes enhance resource sustainability and ethical use of blood products. | 92 | 8 | / | / | / | 100 | ✓ |
| **12** | Disease- and specialty-specific protocols are needed to consistently implement PBM programmes. | 77 | 23 | / | / | / | 100 | ✓ |
| **13** | Key challenges and barriers towards the implementation of PBM include hospital culture, inadequate staff awareness, poor interdisciplinary communication or collaboration, absence of electronic monitoring systems, and resource limitations (staff, time, and finances). | 77 | 23 | / | / | / | 100 | ✓ |

*Response options: A, accept completely; B, accept with some reservation; C, accept with major reservation; D, reject with reservation; and E, reject completely.
